# Using the 11-item Version of the RCADS to Identify Anxiety and Depressive Disorders in Adolescents

**DOI:** 10.1007/s10802-021-00817-w

**Published:** 2021-04-01

**Authors:** Jerica Radez, Polly Waite, Bruce Chorpita, Cathy Creswell, Faith Orchard, Ray Percy, Susan H. Spence, Tessa Reardon

**Affiliations:** 1 School of Psychology and Clinical Language Sciences, University of Reading, Reading, RG6 6AL UK; 2 The Oxford Institute of Clinical Psychology Training and Research, University of Oxford and Oxford Health NHS Foundation Trust, Oxford, OX3 7JX UK; 3 Departments of Experimental Psychology and Psychiatry, University of Oxford, Oxford, OX2 6GG UK; 4 Department of Psychology, University of California, Box 951563, Los Angeles, CA 90095 USA; 5 School of Psychology, University of Sussex, Brighton, BN1 9RH UK; 6 School of Applied Psychology and Australian Institute of Suicide Research and Prevention, Griffith University, Brisbane, QLD 4121 Australia

**Keywords:** Anxiety, Depression, Screening, Questionnaire development, Adolescents

## Abstract

The purpose of this study was to identify items from the Revised Children’s Anxiety and Depression Scale – RCADS-C/P that provided a brief, reliable and valid screen for anxiety and/or depressive disorders in adolescents. In addition, we examined whether adding items assessing suicidal ideation (Moods and Feelings Questionnaire – MFQ- C/P) and symptom impact and duration (items adapted from the Strengths and Difficulties Questionnaire – SDQ) improved the identification of adolescents with anxiety and/or depressive disorders. We compared two samples of adolescents and their parents – a community sample, recruited through secondary schools in England (n = 214) and a clinic-referred sample, who met diagnostic criteria for anxiety and/or depressive disorder and were recruited through a university-based research clinic (n = 246). Participants completed the RCADS-C/P with additional symptom impact and duration items, and the MFQ-C/P. Using ROC curve analyses, we identified a set of 11 RCADS-C/P items (6 addressing anxiety and 5 depression symptoms) for adolescent- and parent-report. This set of 11 symptom items achieved sensitivity/specificity values > .75, which were comparable to corresponding values for the RCADS-47-C/P. Combining adolescent and parent-report improved the identification of anxiety/depression in adolescents compared to using adolescent-report alone. Finally, adding two symptom impact items further improved the sensitivity/specificity of the 11 symptom items, whereas adding suicidal ideation items did not. The 11 RCADS items accurately discriminated between the community and clinic-referred sample with anxiety and/or depressive disorders and have the potential to quickly and accurately identify adolescents with these disorders in community settings.

**Supplementary Information:**

The online version contains supplementary material available at 10.1007/s10802-021-00817-w.

Anxiety and depressive disorders are the most common mental health disorders in adolescents. The estimated prevalence of anxiety disorders in this age group is around 8% (Lawrence et al., 2015; Polanczyk et al., 2015; Sadler et al., 2018) and depressive disorders around 5% (Merikangas et al., 2010). In addition, these disorders often occur simultaneously in adolescents (Axelson & Birmaher, 2001; Essau, 2008). Anxiety and depressive disorders in adolescents are associated with poor academic, social and health outcomes (Lawrence et al., 2015; Riegler et al., 2017; Sadler et al., 2018) and are key contributors to the global economic burden of disease (Whiteford et al., 2013), emphasising the need for early identification and treatment. However, less than two-thirds of young people and their families access any professional help, and only a minority of young people access specialist mental health support (Johnson et al., 2016; Merikangas et al., 2010; Sadler et al., 2018). Key reasons underlying poor treatment utilisation relate to difficulties identifying common mental health problems and the availability of professional help (Lawrence et al., 2015; Sadler et al., 2018).

Professionals within schools and primary care services are well placed to identify symptoms of anxiety and depression in adolescents at an early stage (Department of Health and Department of Education, 2017; Siu & US Preventive Services Task Force, 2016). The availability of questionnaire tools that are able to accurately identify adolescents with anxiety and depressive disorders could help address barriers related to identification in these settings. However, as practitioners working in these settings face significant time restraints, any identification tools must be brief (i.e. < 15 items) (Dowdy et al., 2010), easy to use and interpret (e.g. with clear instructions and cut-off scores) (Glover & Albers, 2007; Myers & Winters, 2002), and psychometrically adequate (e.g. sensitivity/specificity > 75%) (Glover & Albers, 2007). Brief tools for detecting anxiety and depression in adults, such as the GAD-7 (Spitzer et al., 2006) and the PHQ-9 (Kroenke et al., 2001), provide evidence that brief questionnaires can demonstrate good (> 0.80) sensitivity and specificity in primary care settings. However, these questionnaires were developed for adults, making them less appropriate for use with adolescents (Myers & Winters, 2002). Brief anxiety questionnaires for children exist (e.g. SCAS-8; Reardon et al., 2017), but these have only been developed and tested with preadolescent children and do not include depression items, which makes them less suitable for adolescents. Similarly, evaluations of the most often used depression screening questionnaires, such as Short Mood and Feelings Questionnaire (SMFQ; Angold et al., 1995) have not distinguished between preadolescent children and adolescents, which is problematic as adolescents can experience different depressive symptoms to preadolescent children (Baptista et al., 2017). Finally, although adolescents generally provide reliable assessments of their mental health, especially emotional disorders (Aebi et al., 2017; Deighton et al., 2014), a combination of adolescent- and parent-report can provide the most reliable and valid information about adolescents’ mental health difficulties (Becker et al., 2004; Kuhn et al., 2017). Indeed, research suggest that parents and young people focus on symptoms experienced in different contexts (e.g. home environment, at school, with friends) (De Los Reyes et al., 2015), and therefore, a brief identification tool should be available in both, an adolescent-report and parent-report form to provide the most comprehensive assessment of adolescents’ anxiety/depressive symptoms. To our knowledge, there is currently no questionnaire measure of anxiety and depression symptoms in adolescents meeting the above criteria.

One of the most commonly used measures of anxiety and depressive disorder symptoms across the world is the Revised Children's Anxiety and Depression Scale (RCADS; Chorpita et al., 2000). The original 47-item and the shortened 25-item RCADS (Ebesutani et al., 2012) are questionnaire measures of adolescent- and parent-reported symptoms of anxiety and depression in children aged 8 to 18 years. Both RCADS questionnaires demonstrate robust internal consistency in different settings and countries (Piqueras et al., 2017) and are successful in discriminating between clinical samples of young people with a diagnosis of an anxiety disorder or depressive disorder and community samples (Chorpita et al., 2005; Ebesutani et al., 2010, 2017). However, the original RCADS and RCADS-25 are 1) > 15 items, and 2) consistent with DSM-IV, rather than DSM-5 diagnostic criteria (American Psychiatric Association, 2013) (e.g. including OCD items), and do not consider either 3) adolescents’ suicidal ideation, which is a common symptom of depression in adolescents (Orchard et al., 2017), or 4) the impact or duration of anxiety/depression symptoms on adolescents’ lives, which may provide a more reliable estimate of emotional disorders than items that only assess the presence of symptoms (Evans et al., 2017; Goodman, 2001).

The purpose of this study was to identify a brief set of RCADS-C/P items to detect anxiety and depressive disorders in young people aged 11 to 17 years. The study involved a community sample (*n* = 214), and a clinic-referred sample (*n* = 246) who met diagnostic criteria for an anxiety disorder (*n* = 230), and/or a depressive disorder (*n* = 81). We identified a subset of anxiety items from a pool of 31 items from the RCADS anxiety subscale that fit with DSM-5 anxiety disorder symptoms, and a subset of depression items from 10 RCADS depression subscale items (Chorpita et al., 2000). In addition, we set out to determine 1) if adding items that assess suicidal ideation improves the ability of the depression item subset to discriminate between the community sample and clinic-referred with a depressive disorder diagnosis, 2) if adding items associated with symptom impact and duration improves the ability of the total brief item set to discriminate between the community sample and clinic-referred sample with any anxiety/depressive disorder diagnosis, and 3) whether using a combination of reporters (i.e. adolescent-report and parent-report) provides more accurate identification of adolescents with an anxiety/depressive disorder diagnosis compared to adolescent-report alone. Finally, we evaluated the internal consistency, criterion, convergent and divergent validity, and identified optimal cut-off scores for the final brief item set in terms of 1) anxiety score 2) depression score and 3) total score, and compared these psychometric properties with corresponding properties for the original RCADS and the RCADS-25.

## Method

### Ethical Approval

The community sample was recruited as a part of the wider research project on improving access to treatment for anxiety and depressive disorders in adolescents. This project was approved by the University of Reading Research Ethics Committee (UREC 18/28). Permission to retrospectively use clinical data collected from the clinic-referred sample was obtained from the Berkshire Healthcare NHS Foundation Trust (project number 5491).

### Participants

The demographic and clinical characteristics of the community and clinic-referred samples are outlined in Table 1.

**Table 1:** The demographic and clinical characteristics of the community and clinic-referred samples

	Community Sample	Clinic-referred Sample			Statistic (clinic-referred vs community)
Sample Characteristic		Total Sample	Anxiety subsample	Depression subsample	
*N*	214	246	230	81	
Age, Mean (*SD*)	13.63 (0.75)	14.33 (1.73)	14.25 (1.72)	15.11 (1.36)	*t*(458) = 4.313, *p* < 0.01, *d* = 0.40
Gender					
Females, *n* (%)	123 (57.4%)	189 (76.8%)	177 (77.0%)	64 (79.0%)	χ^2^(1) = 18.764, *p* < 0.01, *V* = 0.20
Ethnicity					
White-British, *n* (%)	172 (80.8%)	170 (86.3%)^a^	159 (85.9%)^b^	55 (85.9%)^c^	χ^2^(1) = 1.890, *p* = 0.169
Family Socioeconomic status					
Higher/professional^d^, *n* (%)	100 (46.7%)	98 (50.5%)^f^	92 (51.1%)^g^	34 (52.3%)^h^	χ^2^(1) = 2.258, *p* = 0.323
Parent reporter					
Mother, *n* (%)	186 (86.9%)	204 (88.7%)^i^	190 (88.4%)^j^	64 (85.3%)^k^	
Anxiety/depressive disorder diagnosis^e^, *n* (%)					
Social Anxiety Disorder	-	171 (69.5%)	171 (74.3%)	56 (69.1%)	
Generalised Anxiety Disorder	-	156 (63.4%)	156 (67.8%)	44 (54.3%)	
Major Depressive Disorder	-	73 (29.7%)	60 (26.1%)	73 (90.1%)	
Specific Phobia	-	52 (21.1%)	52 (22.6%)	9 (11.1%)	
Agoraphobia	-	27 (11.0%)	27 (11.7%)	7 (8.6%)	
Separation Anxiety Disorder	-	21 (8.5%)	15 (6.5%)	6 (7.4%)	
Panic Disorder	-	27 (11.0%)	27 (11.7%)	6 (7.4%)	
Dysthymia	-	16 (6.5%)	13 (5.7%)	16 (19.8%)	
Illness Anxiety Disorder	-	3 (1.2%)	3 (1.3%)	0 (0%)	
Unspecified Depressive Disorder	-	2 (0.8%)	1 (0.4%)	2 (2.5%)	
Unspecified Anxiety Disorder	-	2 (0.8%)	2 (0.9%)	0 (0%)	
Primary diagnosis CSR, Mean (*SD*)	-	5.96 (1.00)	5.94 (1.04)	6.46 (0.87)	
Any Anxiety disorder diagnosis, *n* (%)	-	230 (93.5%)	230 (100%)	65 (80.2%)	
Any Depressive disorder diagnosis, *n* (%)	-	81 (32.9%)	65 (28.3%)	81 (100%)	
Non-anxiety/depressive disorder diagnosis (OCD, ODD, PTSD, ADD, ADHD), *n* (%)	-	26 (10.6%)	26 (11.3%)	11 (13.6%)	

^a,b,c^percentage of 197^a^, 180^b^ and 62^c^ parents who provided child ethnicity information

^d^Higher/professional managers, directors, senior officials and professional occupations

^e^anywhere in the diagnostic profile

^f, g, h^percentage of 194^f^, 180^ g^ and 65^h^ parents who provided education/occupation information

^i, j, k^percentage of 230^i^, 215^j^ and 75^k^ parents who provided parent relationship information

*CSR* clinical severity rating on the Anxiety Disorders Interview Schedule (ADIS) and/or Kiddie Schedule for Affective Disorders and Schizophrenia (K-SADS), *OCD* obsessive–compulsive disorder, *ADHD* attention deficit hyperactivity disorder, *ODD* oppositional defiant disorder

#### Community Sample

In total, 1,165 students were screened for anxiety and depression (see Measures section) through two secondary schools in Berkshire, England. Only adolescents for whom both adolescent- and parent-report questionnaire measures were provided were included in this study (*n* = 214). Nearly 70% of adolescents in the community sample were aged between 11 and 14 (classified as ‘younger adolescents’) and 30.8% were aged between 15 and 18 (classified as ‘older adolescents’). Compared to adolescents without parent-report, adolescents whose parents completed the questionnaires were more likely to be female (χ^2^(1) = 11.087, *p* < 0.01, V = 0.11), White-British (χ^2^(1) = 9.255, *p* < 0.01, V = 0.10), and on average scored higher on the RCADS total scale (t(1156) = 3.881, *p* < 0.01, *d* = 0.29) and anxiety subscale (*t*(1157) = 4.313, *p* < 0.01, *d* = 0.32), but not on depression subscale (*t*(1158) = 1.884, *p* = 0.060). However, the effect sizes for these differences were small and there were no significant differences between the samples in relation to other demographic variables (e.g. age, SEN status, English as an additional language).

#### Clinic-referred Sample

The clinic-referred sample was recruited through the Anxiety and Depression in Young People (AnDY) Research Clinic, based at the University of Reading and funded by East Berkshire and Berkshire West Clinical Commissioning Groups. The clinic-referred sample (*n* = 246) included young people aged between 11 and 17, who took part in the standardised diagnostic assessment (see Measures section), and met criteria for a current DSM-5 anxiety and/or depressive disorder anywhere in their diagnostic profile (i.e. primary and secondary diagnoses). A total of 246 adolescents met the study inclusion criteria, of whom 230 met the diagnostic criteria for an anxiety disorder (clinic-referred anxiety subsample) and 81 met the diagnostic criteria for a depressive disorder (clinic-referred depression subsample) anywhere in their diagnostic profile. In the total clinic-referred sample, 48% of adolescents were ‘younger adolescents’ and 52% were ‘older adolescents’.

### Procedure

#### Community Sample

We approached 31 state secondary schools in Berkshire, Buckinghamshire, London, North England and Oxfordshire from September to November 2018. Invited schools had no academic entry criteria and were not taking part in other University research projects. Two large mixed state schools (both in Berkshire) agreed to take part. Participating schools distributed information leaflets and opt-out consent forms to 1,706 parents/carers. After two weeks, the lead researcher (JR) administered paper forms of the adolescent-report questionnaires with students from Year 7 to Year 13, whose parents/carers had not opted out (97.7%). Of 1,237 students invited to take part, 94.2% provided consent/assent and completed the questionnaires. School staff and the researcher oversaw questionnaire completion in classrooms and ensured that responses were confidential. Adolescent background information (e.g. date of birth and gender) was collected from school records at the same time. Where questionnaire responses indicated any risk or raised concerns about the young person’s safety, this was reported to the schools’ safeguarding teams immediately. Both schools then distributed questionnaires to parents/carers, usually by emailing a link to online versions of the questionnaires, and in some cases they also sent paper copies. Up to five reminders within a five-week time frame were then sent to young people’s parents/carers to encourage them to complete the questionnaires. Of 1,165 students who completed self-report questionnaires, 214 (18.7%) parents/carers completed the parent questionnaire as well. To help encourage parent/carer participation, schools were reimbursed £3 for each returned paired (adolescent-parent) questionnaire set.

#### Clinic-referred Sample

Adolescents in the clinic-referred sample were assessed with standardised diagnostic assessments (ADIS-C/P and K-SADS) from January 2017 to June 2019 in the AnDY Research Clinic, University of Reading. At the point of the initial assessment, each young person and their parent completed the RCADS-C/P questionnaire measures. The routine initial assessment questionnaire pack has included symptom impact and duration questions since May 2017, and the Mood and Feelings Questionnaire (MFQ) since March 2018.

### Measures

#### Questionnaires

*Revised Child Anxiety and Depression Scale, Child and Parent Versions (RCADS-C/P; *Chorpita et al., 2000*).* The RCADS is a 47-item questionnaire measure of symptoms of anxiety and low mood in young people, aged from 8 to 18 years. It was developed as an adaptation of the Spence Children’s Anxiety Scale (SCAS; Spence, 1997, 1998) with additional items to assess symptoms of depression. The questionnaire consists of six subscales: separation anxiety disorder (SAD), social phobia (SP), obsessive–compulsive disorder (OCD), panic disorder (PD), generalised anxiety disorder (GAD), and major depressive disorder (MDD). Respondents rate how often each item applies to them/their child, using a 4-point scale from 0 (‘never’) to 3 (‘always’). The RCADS is available in adolescent- and parent-report form and it takes between 10 and 15 min to complete (Chorpita et al., 2000). In the current study, the RCADS anxiety scale (37 items), depression scale (10 items) and total scale (47 items) scores were calculated by summing responses to corresponding items. As OCD is no longer classified as an anxiety disorder in the DSM-5 (American Psychiatric Association, 2013) these six items were not considered for inclusion in the final screening items, and were excluded from the individual item analyses.

*Symptom impact and duration questions.* Participants completed an additional seven questions designed for this study to measure the duration of anxiety/depression symptoms (0 = ‘less than a month’, 1 = ‘1–5 months’, 2 = ‘6–12 months’, 3 = ‘over a year’) and the degree of interference with activities at home/school/friends/outside the school caused by any endorsed symptoms (0 = ‘not at all’, 1 = ‘only a little’, 2 = ‘quite a lot’, 3 = ‘a great deal’). The content of these questions was informed by other similar questionnaires, including the Strengths and Difficulties Questionnaire (SDQ)-Impact Supplement (Goodman, 1999). The individual item score (from 0 to 3) was calculated for each symptom duration/impact item.

*Moods and Feelings Questionnaire (MFQ-C/P; *Angold & Costello, 1987*).* The MFQ is a 33-item (34-item for parent version) screening tool for depression in children and young people, aged between 6 and 17. Respondents are asked to report how they have been feeling or acting in the past two weeks. For each item, they can respond with ‘not true’ (0), ‘sometimes’ (1) or ‘true’ (2). The MFQ total score is calculated by summing participants’ responses to all items. In the present study, we used the MFQ total score and individual item scores for four items assessing young people’s suicidal ideation (‘thought about killing self’, ‘thought about death or dying’, ‘thought family would be better off without self’ and ‘thought life was not worth living’). The utility of these four items to accurately identify adolescents with suicidal ideation has been established in previous research (Hammerton et al., 2014). For the current study, participants’ responses to each of these four items were transformed to a scale from 0 to 3 using linear transformation (Jonge et al., 2014) to match the RCADS response scale. These transformed individual item scores (0, 1.5 or 3) were used in individual item analyses. Prior to transforming individual items, MFQ total scores were calculated by summing participants’ original responses (0 to 2) to all MFQ items.

#### Diagnostic Interviews

The following diagnostic interviews were administered with the clinic-referred sample to assess the presence of an anxiety and/or depressive disorder in young people. Assessors were psychology graduates specifically trained to deliver the diagnostic assessments. All assessments were discussed with an experienced member of the assessment team to agree on a consensus diagnosis. Inter-rater reliability for the presence of an anxiety diagnosis on the ADIS-C/P κ = 1.00 and CSR ICC = 0.93, and of a K-SADS depression diagnoses was κ = 1.00.

*Anxiety Disorder Interview Schedule – Child-Parent Version (ADIS-IV-C/P; *Albano & Silverman, 1996*).*The ADIS-IV-C/P is based on the DSM-IV-TR (American Psychiatric Association, 2000) and consists of two semi-structured interviews (separately with the adolescent and their parent) designed to assess anxiety and other disorders in children and adolescents aged 7 − 16. In the present study, the anxiety sections of the ADIS-IV-C/P were used to determine whether the adolescent met diagnostic criteria for any anxiety disorder. Minor adaptations to the interview schedule were made so the diagnoses were assigned based on the DSM-5. If the adolescent met symptom criteria for a diagnosis, based on either their report or that of their parent, then the clinician would assign a Clinician Severity Rating (CSR), ranging from 0 to 8; a CSR of 4 or more would indicate that the young person met criteria for diagnosis. The diagnosis with the highest CSR was considered to be the primary diagnosis. Studies using the ADIS-IV-C/P provide strong empirical support for its good test–retest reliability with reliability coefficients ranging from 0.78 to 0.99 for child interview and 0.52 to 0.99 for parent interview (Silverman et al., 2001) and high levels (κ > 0.80 for principal diagnosis) of inter-rater agreement (Lyneham et al., 2007). The concurrent validity of the diagnostic tool is especially good for the anxiety section (Wood et al., 2002). As such, ADIS-IV-C/P has been considered as a ‘gold standard’ measure of anxiety disorders in young people’s clinical research.

*Kiddie Schedule for Affective Disorders and Schizophrenia – Present and Lifetime Version (K-SADS-PL DSM-5; *Kaufman et al., 2016*).*The K-SADS-PL is a semi-structured integrated parent and child interview for affective disorders and schizophrenia. In the current study, a DSM-5 (APA, 1994) version of the K-SADS was used. The administration of the interview lasts approximately 30 min with each respondent, and young people and caregivers are interviewed separately. Research studies support adequate psychometric characteristics of the K-SADS-PL with high interrater and test–retest reliability (reliability coefficients ranging form 0.63 to 1 for present and 0.55 to 1 for lifetime diagnoses) and well supported concurrent validity with other existing measures (e.g. Child Behaviour Checklist – CBCL) (Kaufman, 1997). The K-SADS-PL is more widely used in clinical research focused on depression than the ADIS-C/P (Spence, 2018), and in the present study, the depression and mania sections of the K-SADS-PL child and parent interview were used to determine the presence/absence of depressive disorders in adolescents. As per a standard procedure, the diagnosis of depressive disorder was assigned based on adolescent- and parent report combined. In addition, CSR scores were assigned in a similar way as the ADIS-C/P to provide a comparable estimate of the symptom severity/interference.

### Data Analytical Strategy

#### Sample Size Calculation

We computed an a priori power analysis for a Receiver Operating Characteristics (ROC) curve using R, package ‘pROC’ (Robin et al., 2011). Power analysis determined a minimum sample size of 30 participants in each group included in ROC analyses (i.e. participants from the community sample, clinic-referred anxiety subsample and clinic-referred depression subsample) to achieve a sufficient power of 0.80 with an Area Under the Curve (AUC) of 0.70 and α = 0.05. The following procedure was used to identify items for inclusion in the brief screen for anxiety and depression (adolescent- and parent-report versions):

#### Symptom Item Reduction

The pool of potential anxiety items consisted of 31 anxiety items from the RCADS anxiety subscale (i.e. all anxiety items excluding the OCD items), and the pool of potential depression items consisted of 10 depression items from the RCADS depression subscale. To examine the functioning of potential anxiety items, we combined the community sample (*n* = 214) and the clinic-referred anxiety subsample (*n* = 230). Similarly, we examined the functioning of potential depression items by combining the community sample (*n* = 214) with the clinic-referred depression subsample (*n* = 81). We performed the following analyses to reduce the pool of eligible items for adolescent- and parent-report separately: 1) we examined item-total score correlations (anxiety: item-RCADS-C/P anxiety total correlation; depression: item-RCADS-C/P depression total correlation), 2) we calculated the item-discrimination indices using point biserial correlation coefficient (anxiety: item-presence of an anxiety disorder correlation, depression: item-presence of a depressive disorder correlation). In addition, we calculated partial correlations by controlling item-discrimination indices for participants’ gender (female/male) and age group (‘early’ [11–14 years] and ‘late’ [15–17 years] adolescence), to ensure that only items that were able to discriminate between the clinic-referred and community samples across gender and age groups were selected, 3) we investigated the number of missing values. Items with either high (> 0.70) item-total score correlation or high (> 0.30) item-discrimination index, and with a low (< 10%) proportion of missing values were further considered.

#### Examining the Functioning of Alternative Subsets of RCADS Items

First, we created alternative adolescent-report anxiety/depression brief item sets by removing eligible items with the lowest item-discrimination indices one by one. We also considered the content of the items to minimise overlapping content among items, and removed items where the meaning was very similar to an alternative item. We then used ROC curve analyses to compare the ability of 1) alternative subsets of RCADS anxiety items to discriminate between the community sample and the clinic-referred anxiety subsample, and 2) alternative subsets of RCADS depression items to discriminate between the community sample and the clinic-referred depression subsample. Following previous research using similar methodology (e.g. Reardon et al., 2017) we set a threshold value of AUC = 0.7. As the item sets were identified for screening purposes, sensitivity of the cut-off score was prioritised over specificity (Kraemer, 1992). The cut-off scores with sensitivity of > 0.8 and specificity of > 0.7 were calculated. Where sensitivity/specificity > 0.8/0.7 were not achievable, cut-off scores with sensitivity/specificity > 0.7/0.7 or > 0.7/0.6 were considered. We repeated the ROC analyses across different gender (female/male) and age (‘early’ [11–14 years] and ‘late’ [15–17 years] adolescence) groups to identify item sets that performed similarly across gender/age troups.

Once we had identified the brief set of adolescent-report anxiety items and depression items, we then examined the functioning of alternative parent-report anxiety/depression item combinations using the same ROC analyses. Given the practical utility of including common items across adolescent- and parent-report, firstly we examined the functioning of parent-report anxiety/depression brief item sets that included the same items as the final combinations for adolescent-report. Then, we created alternative parent-report anxiety/depression item combinations, using the same procedure as we used for adolescent-report (i.e. by removing eligible parent-report items with the lowest item-discrimination indices one by one) to identify the ‘optimal’ set of parent-report anxiety/depression items, and compared these to parent-report item sets that included the same items as those identified for adolescent-report.

#### Examining the Functioning of Items Assessing Suicidal Ideation and Symptom Impact and Duration Items and Whether Adding them Improves the Measures

We calculated item-discrimination indices using point biserial correlation coefficients for the 1) items assessing young people’s suicidal ideation and the presence of depressive disorder diagnosis, and 2) symptom impact and duration items and the presence of any anxiety/depressive disorder. Items with a high (> 0.30) item-discrimination index, and a low (< 10%) proportion of missing values were further considered. Similar to the process of identifying brief subsets of symptom items, we then used ROC analyses to identify items assessing 1) suicidal ideation and 2) symptom impact/duration, to use together with the brief sets of symptom items. Suicidal ideation/impact and duration items with the lowest item-discrimination indices were removed one by one. We then examined the functioning of parent-report brief depression/total symptom item sets together with the same suidical ideation/symptom impact and duration items that were identified in adolescent-report. The same procedure (i.e. by removing items with the lowest item-discrimination indices one by one) was then repeated for the parent-report to identify the optimal combinations of suicidal ideation/impact and duration items to use together with the brief depression/total symptom item sets for parent-report. We then compared these to using the same suicidal ideation/symptom impact and duration items as the adolescent-report. Finally, we used a series of logistic regressions to establish whether adding the identified combination of 1) suicidal ideation items improved the ability of the brief depression screen to discriminate between adolescents in the community and clinic-referred depression subsample, and 2) symptom impact and duration items improved the ability of the brief total screen to discriminate between adolescents in the community and clinic-referred sample. Independent variables were added in logistic regression model one by one, starting with the brief depression/total symptoms item set.

#### Combining Adolescent-report and Parent-report

We examined whether combining adolescent- and parent-report improved the identification of adolescents with an anxiety/depressive disorder diagnosis, compared to using adolescent-report alone. We performed logistic regressions with the adolescent group (i.e. community sample and clinic-referred anxiety/depression subsample) as the dependent variable and different combination of participants’ responses (i.e. adolescent-report and adolescent-report + parent-report) as independent variables. Participants’ responses were added to the regression models one by one. As adolescent self-reported anxiety/depressive symptoms were a primary focus of the current study, we firstly included adolescent-report responses in the logistic regression models.

#### Psychometric Evaluation of the Brief set of RCADS Anxiety and Depression Item Sets and Comparison with the Original RCADS and the RCADS-25

Using the brief set of RCADS items, we calculated adolescent- and parent-report brief anxiety, depression and total scores for all participants by summing their responses to corresponding items. We then calculated the following psychometric properties of the brief adolescent- and parent-report anxiety/depression/total scores, and compared these with the corresponding properties of the original RCADS and RCADS-25 anxiety/depression/total scores: internal consistency, convergent, discriminant, and criterion validity. Internal consistency was calculated using McDonald's omega coefficients. We assessed convergent validity using Pearson correlation coefficients between the brief depression score/original RCADS depression score/RCADS-25 depression score and MFQ total score, and divergent validity using Pearson correlation coefficients between the brief anxiety score/original RCADS anxiety score/RCADS-25 anxiety score and MFQ total scores. Criterion validity of the brief anxiety/depression/total score, and original RCADS/RCADS-25 anxiety/depression/total scores was assessed using ROC curve analyses following procedures described previously, to identify the AUC and optimal cut-off scores and their corresponding sensitivity/specificity values for the optimal cut-off scores. ROC curve analyses were repeated for separate gender and age groups.

### Missing Data

Across all items, missing responses were < 4% in the community sample and < 14% in the clinic-referred sample, with the exception of MFQ-C/P and symptom impact items and duration items in the clinic referred sample (29–43%), which as detailed above were only introduced into the routine initial assessment in May 2017 (symptom impact and duration items) and March 2018 (MFQ-C/P). Following previous research (e.g. Donnelly et al., 2019), we handled all the missing values as pairwise missing. We performed sensitivity analyses to confirm that the clinic-referred sample of adolescents without MFQ-C/P responses was not significantly different from the clinic-referred sample of adolescents who had fully completed the questionnaires. Mean RCADS-C/P anxiety and depression scores were calculated for participants in the clinic-referred sample who had fully completed the questionnaires and for the participants that had only completed RCADS-C/P. Means were compared using independent samples *t-*tests. In addition, we calculated Pearsons's correlation coefficients between RCADS anxiety and depression subscales for participants with fully and partially completed questionnaires. No significant differences were found between the two groups of adolescents, confirming that these data can be treated as missing completely at random (MCAR). Due to the large sample sizes (> 200), we used a conservative *p*-value of 0.01. All the analyses were performed using R version 3.6.1 (R Core Team, 2019) packages ‘cvAUC’ (LeDell et al., 2014), ‘pROC’ (Robin et al., 2011), ‘psych’ (Revelle, 2018) and ‘userfriendlyscience’ (Peters, 2018).

## Results

### Symptom Item Reduction

Item-anxiety/depression total correlations and item-discrimination indices (total; controlled for adolescents’ gender and age) for adolescent-report are displayed in Table 2. Corresponding item-total correlations and item-discrimination indices for parent-report are provided in Electronic Supplementary Material 1.

**Table 2:** Rank-ordered item-total correlations and item-discrimination indices for the RCADS-C items

			Item-anxiety/depression diagnosis correlation		
Item number	Item (RCADS Subscale)	Item-total	Total	Controlled for Gender	Controlled for age
	Anxiety items				
RCADS35^a,b^	I worry about what is going to happen (GAD)	0.76*	0.38*	0.35*	0.37*
RCADS28^a,b^	When I have a problem, I feel shaky (PD)	0.72*	0.32*	0.28*	0.31*
RCADS41^a,b^	I worry that I will suddenly get a scared feeling when there is nothing to be afraid of (PD)	0.71*	0.31*	0.27*	0.29*
RCADS22^a,b^	I worry that bad things will happen to me (GAD)	0.71*	0.26*	0.24*	0.27*
RCADS24^a,b^	When I have a problem, my heart beats really fast (PD)	0.71*	0.36*	0.32*	0.35*
RCADS20^a,b^	I worry I might look foolish (SOC)	0.71*	0.35*	0.31*	0.33*
RCADS34^a,b^	All of a sudden I feel really scared for no reason at all (PD)	0.71*	0.37*	0.33*	0.36*
RCADS1^a,b^	I worry about things (GAD)	0.71*	0.31*	0.28*	0.30*
RCADS43^b^	I feel afraid that I will make a fool of myself in front of people (SOC)	0.68*	0.27*	0.24*	0.26*
RCADS14^b^	I suddenly feel as if I can't breathe when there is no reason for this (PD)	0.68*	0.27*	0.24*	0.26*
RCADS27^b^	I worry that something bad will happen to me (GAD)	0.68*	0.25*	0.23*	0.25*
RCADS30^b^	I worry about making mistakes (SOC)	0.67*	0.25*	0.22*	0.24*
RCADS12^b^	I worry that I will do badly at my school work (SOC)	0.65*	0.26*	0.22*	0.24*
RCADS32	I worry what other people think of me (SOC)	0.64*	0.28*	0.24*	0.27*
RCADS7^b^	I feel scared when I have to take a test (SOC)	0.64*	0.26*	0.22*	0.24*
RCADS39^b^	My heart suddenly starts to beat too quickly for no reason (PD)	0.64*	0.26*	0.23*	0.25*
RCADS26	I suddenly start to tremble or shake when there is no reason for this (PD)	0.63*	0.27*	0.24*	0.26*
RCADS8^b^	I feel worried when I think someone is angry with me (SOC)	0.62*	0.26*	0.22*	0.25*
RCADS3^a,b^	When I have a problem, I get a funny feeling in my stomach (PD)	0.61*	0.30*	0.26*	0.29*
RCADS18^a,b^	I have trouble going to school in the mornings because I feel nervous or afraid (SEP)	0.61*	0.47*	0.45*	0.47*
RCADS4^b^	I worry when I think I have done poorly at something (SOC)	0.61*	0.20*	0.17*	0.17*
RCADS45^a,b^	I worry when I go to bed at night (SEP)	0.61*	0.41*	0.38*	0.41*
RCADS33^a,b^	I am afraid of being in crowded places (like shopping centres, the cinema, buses, busy playgrounds) (SEP)	0.60*	0.31*	0.28*	0.30*
RCADS36	I suddenly become dizzy or faint when there is no reason for this (PD)	0.55*	0.20*	0.16*	0.19*
RCADS13	I worry that something awful will happen to someone in my family (GAD)	0.54*	0.07	0.02	0.07
RCADS38^b^	I feel afraid if I have to talk in front of my class (SOC)	0.53*	0.22*	0.18*	0.21*
RCADS37	I think about death (GAD)	0.51*	0.14*	0.12*	0.14*
RCADS46^b^	I would feel scared if I had to stay away from home overnight (SEP)	0.48*	0.26*	0.24*	0.28*
RCADS9	I worry about being away from my parents (SEP)	0.48*	0.10	0.07	0.12
RCADS5	I would feel afraid of being on my own at home (SEP)	0.47*	0.25*	0.22*	0.26*
RCADS17	I feel scared if I have to sleep on my own (SEP)	0.41*	0.18*	0.15*	0.20*
	Depression items				
RCADS29^a,b^	I feel worthless (MDD)	0.81*	0.40*	0.39*	0.38*
RCADS19^a,b^	I have no energy for things (MDD)	0.80*	0.44*	0.43*	0.40*
RCADS40^a,b^	I feel like I don’t want to move (MDD)	0.79*	0.35*	0.33*	0.32*
RCADS2^a,b^	I feel sad or empty (MDD)	0.77*	0.40*	0.39*	0.36*
RCADS21^a,b^	I am tired a lot (MDD)	0.75*	0.40*	0.39*	0.35*
RCADS47^a^	I feel restless (MDD)	0.75*	0.31*	0.30*	0.28*
RCADS6^a,b^	Nothing is much fun anymore (MDD)	0.73*	0.41*	0.41*	0.38*
RCADS25^a,b^	I cannot think clearly (MDD)	0.73*	0.29*	0.28*	0.27*
RCADS11^b^	I have trouble sleeping (MDD)	0.64*	0.28*	0.26*	0.28*
RCADS15^a^	I have problems with my appetite (MDD)	0.64*	0.31*	0.30*	0.29*

*GAD* generalised anxiety disorder, *PD* panic disorder, *SOC* social anxiety disorder, *SEP* separation anxiety disorder

^a^Item retained in the reduced pool of eligible adolescent-report anxiety items

^b^Item retained in the reduced pool of eligible parent-report anxiety items

*p < 0.01

**Table 3:** ROC curve analyses for the 11-items from RCADS-C (current study), the original RCADS-C, and the RCADS-25-C

	RCADS: Anxiety			RCADS: Depression			RCADS: Total		
Number of items	6^a^	37^b^	15^c^	5^a^	10^b^	10^c^	11^a^	47^b^	25^c^
Total									
AUC	0.81	0.74	0.71	0.87	0.86	0.86	0.82	0.76	0.75
Cut-off	7.5	44.5	13.5	8.5	15.5	15.5	12.5	56.1	27
Sensitivity/ Specificity	0.77/0.74	0.70/0.69	0.73/0.61	0.82/0.77	0.76/0.76	0.76/0.76	0.80/0.71	0.71/0.70	0.70/0.68
*n* (positive; negative)	221;226	228;230	229;229	79;366	80;379	80;379	230;204	244;214	245;214
Boys									
AUC	0.85	0.78	0.72	0.93	0.93	0.93	0.87	0.81	0.78
Cut-off	4.5	32.4	10.9	7.5	13.5	13.5	8.5	42.9	21.5
Sensitivity/ Specificity	0.83/0.75	0.74/0.71	0.70/0.62	0.88/0.83	0.88/0.77	0.88/0.77	0.84/0.70	0.75/0.75	0.75/0.74
*n* (positive; negative)	48;95	53;95	53;95	17;126	17;131	17;131	51;87	57;91	57;91
Girls									
AUC	0.77	0.71	0.68	0.84	0.83	0.83	0.77	0.72	0.71
Cut-off	8.5	45.1	15.5	8.5	15.5	15.5	14.5	59.5	28.6
Sensitivity/ Specificity	0.73/0.70	0.74/0.62	0.68/0.61	0.82/0.71	0.75/0.71	0.75/0.71	0.72/0.68	0.70/0.64	0.70/0.62
*n* (positive; negative)	173;131	175;135	176;134	62;240	63;248	63;248	179;117	187;123	188;123
Older adolescents									
AUC	0.82	0.75	0.73	0.83	0.82	0.82	0.84	0.77	0.77
Cut-off	7.5	45.1	14.5	8.5	15.5	15.5	14.5	58.1	28.6
Sensitivity/ Specificity	0.81/0.80	0.71/0.70	0.70/0.65	0.79/0.70	0.73/0.72	0.73/0.72	0.80/0.76	0.73/0.73	0.73/0.73
*n* (positive; negative)	110;77	112;80	113;79	58;131	59;134	59;134	120;63	126;66	127;66
Younger adolescents									
AUC	0.80	0.74	0.70	0.92	0.92	0.92	0.78	0.75	0.73
Cut-off	7.5	41.6	13.5	8.5	16.8	16.8	11.5	51.5	23.5
Sensitivity/ Specificity	0.74/0.72	0.72/0.67	0.72/0.61	0.90/0.80	0.86/0.83	0.86/0.83	0.78/0.66	0.70/0.65	0.68/0.61
*n* (positive; negative)	111;149	116;150	116;150	21;235	21;245	21;245	110;141	118;148	118;148

^a^11-item RCADS-C, ^b^RCADS-C, ^c^RCADS-25-C

### Anxiety Items

All RCADS-C/P anxiety symptom items were significantly (*p* < 0.01) correlated with the original RCADS-C/P anxiety total score, with correlations ranging from 0.41 to 0.76 for adolescent-report items and from 0.43 to 0.81 for parent-report items. With the exception of item 13 (‘I worry that something awful will happen to someone in my family’) and item 9 (‘I worry about being away from my parents’), all RCADS-C anxiety items discriminated between the clinic-referred anxiety subsample and the community sample (*p* < 0.01), and this was maintained after controlling for adolescents’ gender and age. All RCADS-P anxiety items were) associated with adolescents’ group (community versus clinic-referred anxiety subsample; *p* < 0.01), and again, these associations were retained after controlling for adolescents’ gender and age. Twelve RCADS-C anxiety symptom items had an item-total correlation ≥ 0.7 and/or item-discrimination index ≥ 0.3 and were therefore further considered for inclusion the brief RCADS anxiety item set. Notably, 24 RCADS-P items met the same criteria, including parent-report versions of all 12 retained RCADS-C items.

### Depression Items

Correlations between the RCADS-C/P depression items and the original RCADS-C/P depression total score were moderate to large (ranging from 0.60 to 0.81 for adolescent-report and from 0.49 to 0.83 for parent-report, *p* < 0.01). All adolescent and parent-report depression symptom items discriminated between the community and clinic-referred depression subsample, with item discrimination indices ranging from 0.16 to 0.40 for adolescent-report and 0.25 to 0.42 for parent-report. As with the anxiety items, these associations remained significant (*p* < 0.01) for both adolescent- and parent-report after controlling for adolescent gender and age. Nine adolescent-report and 8 parent-report depression symptom items had an item-total correlation ≥ 0.7 and/or item-discrimination index ≥ 0.3 and were retained for further consideration, including seven common items across the reduced adolescent/parent-report item pool.

### Alternative Numbers of Items

Findings from a series of ROC curve analyses examining the functioning of alternative subsets of adolescent-report anxiety items (≤ 12 items) and depression items (≤ 9 items) are displayed in Electronic Supplementary Material 2.^1^

### Anxiety Item Selection

The final subset of adolescent-report RCADS anxiety items consisted of six anxiety symptom items (RCADS-C 18, 45, 35, 34, 24, 20) assessing symptoms associated with separation anxiety disorder, generalised anxiety disorder, panic disorder and social anxiety disorder. The set of 6-items identified adolescents in the clinic-referred anxiety subsample with an AUC of 0.81 and using an optimal cut-off score of 7.5, achieved sensitivity/specificity values of 0.77/0.74. Parent-report on the same 6 anxiety items achieved an AUC of 0.86, and the optimal cut-off of 5.5 was associated with sensitivity/specificity values of 0.80/0.70.

### Depression Item Selection

The final subset of adolescent-report RCADS depression items consisted of five depressive symptoms items (RCADS 19, 6, 29, 2, 21), reflecting a lack of energy/fatigue, anhedonia, feelings of worthlessness and depressed mood. The 5 items identified adolescents in the clinic-referred depression subsample with an AUC of 0.89 and an optimal cut-off score of 9.25 was associated with sensitivity/specificity values of 0.83/0.79. Parent-report using the same 5 depression items as identified for adolescents achieved an AUC of 0.87 and an optimal cut-off score of 6.75, with sensitivity/specificity values of 0.90/0.77 respectively.

### Examining the Functioning of Items Assessing Risk of Suicide/Self-harm and Symptom Impact and Duration

Electronic Supplementary Material 3 displays 1) the rank ordered item discrimination indices for items assessing suicidal ideation and symptom impact and duration, 2) findings from the ROC curve analyses using alternative combinations of suidical ideation/impact and duration items together with the 5 RCADS depression items/11 RCADS items and 3) a series of binary logistic regressions using either adolescent-report or parent-report, with and without additional items assessing suicidal ideation/impact/duration.

Adding one item assessing suicidal ideation (MFQ 19-C/P ‘I/My child thought about killing myself/himself/herself’) to the 5 RCADS depression items achieved AUC values of > 0.80, and sensitivity/specificity values of 83/0.79 (adolescent-report) and 0.90/0.77 (parent-report). However, the results of the binary logistic regressions illustrated that this item did not make a significant contribution to the identification of adolescents in the clinic-referred depression sample (adolescent-report: χ^2^(1) = 6.23, *p* = 0.013, parent-report: χ^2^(1) = 0.451, *p* = 0.502).

Using two of the symptom impact items (‘How much do these difficulties upset or distress you/your child?’ and 'How much do these difficulties get in the way of your/your child's everyday life at school?’) in combination with the 11 RCADS items achieved AUC values of 0.82-0.90, with optimal cut-off total scores associated with sensitivity/specificity values of 0.84/0.72 and 0.82/0.80 for adolescent-report and parent-report respectively. The results of binary logistic regressions showed that adding these two impact items improved the overall identification of adolescents in the clinic-referred sample, using both adolescent-report (χ^2^(1) = 26.59, *p* < 0.01) and parent-report (χ^2^(1) = 17.06, *p* < 0.01). Notably, the two impact questions better predicted whether adolescents were in the clinic-referred sample or the community sample than the symptom items (odds ratio [OR] 1.12 and 1.16, compared to 1.85 and 1.69, for adolescent- and parent-report respectively).

### Combining Respondents

Both adolescent- and parent-report made a significant contribution to identifying adolescents in the clinic-referred anxiety subsample versus the community sample (OR = 1.15 and 1.35, respectively). Adding parent-report improved the overall logistic regression model (χ^2^(1) = 69.08, *p* < 0.01), indicating that a combination of adolescent-report and parent-report provides more accurate identification of adolescents with anxiety disorders than adolescent-report alone. Adolescent-report and parent-report depression items both significantly contributed to accurate identification of depressive disorders (OR = 1.49 and 1.33, respectively, *p* < 0.01). Adding parent-report improved the overall regression model fit (χ^2^(1) = 26.00, *p* < 0.01), indicating that the combination of adolescent- and parent-report leads to the most accurate identification of adolescents with depressive disorders.

### Psychometric Evaluation of the 11 RCADS Items and Comparison with the Original RCADS-47 and the RCADS-25

#### **Internal Consistency:**

The McDonald's omega coefficient for the 11 RCADS items ranged from 0.72-0.88 in the total clinic-referred sample, 0.73-0.88 in the clinic-referred anxiety subsample, 0.70-0.78 in the clinic-referred depression subsample and 0.87-0.94 in the community sample, demonstrating acceptable/good internal consistency. McDonald's omega coefficients with 95% confidence intervals for the 11-item RCADS total score and the 5 item depression score/6 item anxiety score, and the original RCADS and RCADS-25 are outlined in Electronic Supplementary Material 4.

#### **Convergent and Divergent Validity:**

The correlation coefficients between the 5 item depression score and the MFQ-C/P total scores were moderate to high (ranging from 0.63 to 0.80, *p* < 0.01) in the community sample, total clinic-referred sample and both clinic-referred subsamples, demonstrating favourable convergent validity. Similarly, correlations between 6-item anxiety score and MFQ-C/P total scores were at least moderate (0.50 to 0.77, *p* < 0.01) in the community sample, total clinic-referred sample, and clinic-referred anxiety subsample, but weak to moderate (≤ 0.44, *p* > 0.01) in the clinic-referred depression subsample. Corresponding convergent/divergent validity coefficients for the original RCADS and the RCADS-25 were comparable to those for the 11-item RCADS, with similar patterns of associations in each sample (see Electronic Supplementary Material 5).

#### **Criterion validity/ROC curve analyses:**

The AUC and optimal cut-off scores, with corresponding sensitivity/specificity values for the 11-item RCADS, the original RCADS, and the RCADS-25 are outlined in Table 3 (adolescent-report) and Electronic Supplementary Material 6 (parent-report). Analyses are presented for the total sample and subsamples (girls, boys, older and younger adolescents).^2^

The brief adolescent-report for anxiety (6 items)/depression (5 items)/total (11 items) identified adolescents with either an anxiety or depressive disorder with a moderate-to-good level of accuracy, with AUC values > 0.70 (0.77-0.93). The optimal cut-off score for the anxiety items, depression items and total were associated with sensitivity/specificity values > 0.80/0.70 or > 0.70/0.70 in each group, with the exception of the 11-item RCADS total score among adolescent girls (sensitivity/specificity 0.72/0.68) and younger adolescents (sensitivity/specificity 0.78/0.66). The original RCADS and RCADS-25 achieved AUC values > 0.60 (0.68-0.93), although the optimal cut-off scores were associated with more varied sensitivity/specificity values than corresponding values for the 11-item RCADS total score, ranging from 0.68/0.61 and 0.74/0.71 for the RCADS and RCADS-25 anxiety subscales respectively to 0.73/0.72 and 0.86/0.83 for the RCADS and RCADS-25 depression subscales.

The parent-report 11-item RCADS total score discriminated between adolescents in the community sample and adolescents in the clinic-referred sample with a good level of accuracy, with AUC values > 0.80 (0.83 to 0.91). The sensitivity/specificity values > 0.80/0.70 or > 0.70/0.70 were achieved for the parent-report 5 anxiety items, 6 depression items and the 11-item total score in each group, ranging from 0.75/0.75 to 0.84/0.83 for the 6 anxiety items, and from 0.82/0.74 to 0.93/0.75 for the 5 depression items. The original RCADS-P and RCADS-25-P similarly achieved AUC values > 0.80, although notably, the sensitivity/specificity values associated with the optimal cut-off scores on the RCADS-P and RCADS-P-25 did not exceed corresponding values for the 11-item RCADS-P, and this trend was consistent across both age and gender groups.

## Discussion

Brief and accurate screening measures for symptoms of anxiety and depression in adolescents are needed to help identify young people with these commonly occurring mental health problems in community settings, such as schools or primary care services. Consequently, we identified a set of 11 items from the widely-used RCADS to screen for DSM-5 anxiety and depressive disorders symptoms within this specific age range that were able to discriminate between the community sample of adolescents and a clinic-referred sample of adolescents with an anxiety/depressive disorder diagnosis. The study also identified two optional symptom impact questions that further increased the accuracy of 11 RCADS symptom items (items and scoring details are available in Electronic Supplementary Material 7 and on the RCADS authors’ website).

Two additional symptom impact items related to distress and interference at school further improved the accuracy of 11 RCADS adolescent- and parent-report symptom items, and notably, were able to better discriminate between adolescents in the community and clinic-referred sample than the symptom items alone. The superiority of impact items over symptom items when predicting mental health problems in children and adolescents is consistent with previous research (Evans et al., 2017; Goodman, 1999; Stringaris & Goodman, 2013). Items assessing adolescent’s suicidal ideation on the other hand did not improve the accuracy of a brief set of depression items. Although suicidal ideation represents a common characteristic of adolescent depression in clinic-referred samples (e.g. Orchard et al., 2017), large-scale community studies (e.g. Vander Stoep et al., 2009) suggest that suicidal ideation is common in non-help-seeking populations as well with over 60% of adolescents experiencing suicidal thoughts at least once over the course of 18 months. Suicidal ideation, therefore, might not be a key characteristic that distinguishes adolescents in clinic-referred samples from those in community samples.

We found that using both adolescent-report and parent-report of the 11 RCADS items led to the most accurate discrimination between the community and clinic-referred sample of adolescents with anxiety/depressive disorder diagnosis, which is consistent with previous research (e.g. Choudhury et al., 1998; Goodman et al., 2000; Villabø et al., 2012). In discriminating between a clinic-referred depression subsample and community sample, adolescent-report was superior to parent-report. However, perhaps surprisingly within this age group, in discriminating between a clinic-referred anxiety subsample and community sample, parent-report was better than adolescent-report. These findings are consistent with previous studies comparing single informants for identifying anxiety disorders (Reardon et al., 2017) and depression (Lewis et al., 2014) in children and young people. It might be that the parents are more able to detect symptoms of observable behaviours, including anxiety, but not depressive symptoms, which are usually less noticeable (Martel et al., 2017).

Together the 11 RCADS items demonstrated good psychometric properties which were comparable with those of the original RCADS and RCADS-25. Overall, sensitivity/specificity values of the depression and anxiety scores and the total score were at least > 0.70/0.70, which was replicated across different age and gender groups, with the exception of the total score for adolescent girls and younger adolescents (sensitivity/specificity values of 0.72/0.68 and 0.78/0.66, respectively).

The internal consistency of the adolescent- and parent-report 11-item total score and 5-item depression score were good (McDonald's omega coefficients > 0.80) in the community sample, total clinic-referred sample, and clinic-referred anxiety subsample, although the adolescent- and parent-reported 6-item anxiety score demonstrated slightly lower, yet still acceptable, internal consistency values in the clinic-referred samples. Notably, the 11-item RCADS, as well as the original RCADS and RCADS-25 demonstrated better internal consistency in the community sample compared to the clinic-referred sample, consistent with previous research using the original RCADS and RCADS-25 (Piqueras et al., 2017).

The 5 RCADS depression items for adolescent- and parent-report strongly correlated with the MFQ-C/P evidencing convergent validity. Correlation coefficients between the 6 RCADS anxiety items and MFQ-C/P (divergent validity) were lower albeit still moderate and in most cases significant. This lack of divergent validity probably reflects high levels of comorbidity between anxiety and depressive symptoms in adolescents (Cummings et al., 2014; Essau, 2003; Seligman & Ollendick, 1998). Notably, the 11 RCADS items, and the original RCADS/RCADS-25 demonstrated similar patterns of convergent and divergent validity for anxiety/depression scores for adolescent- and parent-report.

### Implications

Due to the brevity, easy administration/scoring, and good levels of sensitivity and specificity, the 11 RCADS items identified in this study have potential for use in community settings, such as schools and primary care, as a measure to screen for anxiety or depressive disorders. For adolescent-report, we recommend using cut-off scores of ≥ 5 for adolescent boys, and ≥ 9 for adolescent girls (anxiety score), ≥ 8 for adolescent boys, and ≥ 9 for adolescent girls (depression score), and ≥ 9 for adolescent boys and ≥ 14 for adolescent girls (total score). Using additional impact questions, the cut-off for the total score increases to ≥ 14 for adolescent boys and ≥ 18 for adolescent girls. When using parent-report, we recommend using cut-offs of ≥ 5 (adolescent boys) and ≥ 7 (adolescent girls) for the anxiety score, ≥ 6 (adolescent boys) and ≥ 7 (adolescent girls) for the depression score, ≥ 8 (adolescent boys) and ≥ 11 (adolescent girls) for total score and ≥ 13 (adolescent boys) and ≥ 15 (adolescent girls) for total score with additional impact items.

To increase the accuracy of 11 RCADS items we recommend using additional symptom impact items. Adolescents’/parents’ responses to these two items should be interpreted in relation to the total score and not in relation to the depression or anxiety symptom scores alone. Our findings suggest that each respondent (i.e. adolescent and parent) makes a significant contribution to the identifying adolescents with anxiety and/or depressive disorders, which warrants the single-informant approach in situations when only adolescent/parent is available. Ideally, the 11-RCADS items plus symptom impact items should be completed by both the adolescent and their parent/carer as this leads to the most accurate identification of young people with anxiety/depression. The items have the potential to be used on an individual basis, such as in primary care, or as a part of universal screening for anxiety and depressive disorders in schools. In particular, the brief set of 11 RCADS items should be considered when the administration and scoring of the 47-/25-item RCADS is not feasible due to logistical and time constraints (e.g. in educational settings). Practitioners might also prioritise a brief set of 11 RCADS items when they do not require information relating to specific anxiety disorders, but are aiming to identify adolescents with any anxiety and/or depressive disorders. In each case, the items should be administered by well-trained health/social care/education professionals, who are familiar with standard procedures (e.g. referral of adolescents to the appropriate services). When administered on a large scale (e.g. universal school-based screening), it will be instrumental that the schools have the resources to manage the screening process, including facilitating support for those adolescents who are in need of and request further support. This could potentially include following-up a sizeable proportion (i.e., up to 30%) of adolescents screened (Kuo et al., 2009). Although the items assessing adolescent’s suicidal ideation are not included in the final brief set of items, it is important that the thorough risk assessment is carried out with adolescents scoring above the threshold for the depression items (National Institute for Health and Care Excellence, 2019).

### Strengths and Limitations

We applied a rigorous methodological process to identify a brief set of RCADS items that are easy to use/interpret, and psychometrically adequate to discriminate between adolescents in a community sample and adolescents with a diagnosis of anxiety and/or depressive disorder diagnosis. The 11-item set from the RCADS was able to discriminate between those two groups of adolescents with comparable accuracy to the original RCADS and RCADS-25. Furthermore, the accuracy of a brief set of 11 RCADS items was comparable to the accuracy of well-established depression (e.g. CDI) and anxiety (e.g. SCARED) screening questionnaires for children and young people (Desousa et al., 2013; Roseman et al., 2016). The 11 items take only a few minutes to be completed, producing clear cut-off scores and the same items are used in both the adolescent- and parent-report versions, meaning items completed by different respondents can be easily compared. As such, the brief set of 11 items has the potential for use in community settings where the primary purpse is to screen for any anxiety or depressive disorder, and administering longer questionnaires may not be feasible.

Nevertheless, our study has several limitations. First, only participants in the clinic-referred sample, and not those in the community sample, were assessed using the standardised diagnostic interviews. Given the prevalence rates of anxiety and depressive disorders in adolescents, some participants in the community sample might have met the criteria for anxiety and/or depressive disorders and were wrongly classified as participants without an anxiety/depressive disorder diagnosis (false negatives). In addition, the two samples were recruited through different methods (i.e. community sample through local secondary schools and clinic-referred sample through university-based clinic), and all participants in the clinic-referred sample were a help-seeking population (i.e. higher percentage of females and higher mean age than in a community sample) which might have contributed to differences between the samples. As the clinic-referred sample was recruited through a specialiased (anxiety and depression) clinic, our findings have limited generalisability to other diagnostically heterogenous clinic-referred samples. Furthermore, we used the same samples to both develop and evaluate items. Unlike the original RCADS, the brief set of 11 items do not provide information about specific anxiety disorders. As our aim was to identify items that were strong predictors of any anxiety disorder, and because there is overlap in symptoms among anxiety disorders, we used a data-driven approach rather than being prescriptive about what items should be included (e.g., having items that reflected symptoms of specific anxiety disorders) in the final item set. Using different approach (e.g. content-driven) would likely result in a different final item set and potentially add to the construct validity of the scale. It is also important to acknowledge that we evaluated the utility of symptom impact and duration items in relation to the original 47-item RCADS, and therefore, the utility of the two identified symptom impact items may vary when presented with a shorted 11-item version of the symptom measure. In addition, the original RCADS and RCADS-25 items were developed through different methodological procedures and for different purposes than this brief screening tool, making it hard to draw direct comparisons between the psychometric characteristics of all three questionnaire measures. Finally, although we used an opt-out approach to collect adolescent-report questionnaires, which is recommended to maximise student participation and increase demographic variability (Eaton et al. 2004; Liu et al., 2017), parent response rates were still low (18.7%). Conequently, the selected community sample differed significantly from the whole community sample in terms of certain demographic (e.g. a higher percentage of adolescent girls) and clinical (e.g. a higher level of reported anxiety symptoms on the RCADS) characteristics. This bias introduces the possibility that the findings might be less generalisable to wider population.

### Future Research

Our findings highlighted several possibilities for future research. First, the screening items should be re-evaluated in a new community sample of adolescents and their parents. Similarly, the utility of the two identified impact items should be re-assessed in relation to the short set of 11 symptom items. Participants in the community sample should be assessed with standardised diagnostic assessments so that the capacity to identify adolescents with and without specific anxiety/depressive disorders in the community can be evaluated. If possible, future research should include larger (> 500) samples of young people and their parents, which would enable researchers to use more novel methodological frameworks, such as Item Response Theory (IRT) (Jiang et al., 2016). Given the high predictive value of the two symptom impact items, future research should also investigate the utility of using these items alone to identify anxiety and/or depressive disorders in young people. If successful this could provide a much more time-efficient means of identification within universal screening systems. In addition, although the sample included in the current study was representative of adolescent population in England, it is unclear how applicable the findings are for ethnic minority groups and further research is required to specifically examine this. Finally, similar to the use of brief anxiety and mood measures to monitor routine clinical outcomes in adult services (Gyani et al., 2013), this brief set of items have the potential to be applied beyond screening purposes, such as to monitor progress through treatment of anxiety and depressive disorders in adolescents and, as such, the items’ sensitivity to change warrants specific examination.

## Supplementary Information

Below is the link to the electronic supplementary material.

Supplementary file1 (PDF 86 KB)

Supplementary file2 (PDF 99 KB)

Supplementary file3 (PDF 162 KB)

Supplementary file4 (PDF 71 KB)

Supplementary file5 (PDF 79 KB)

Supplementary file6 (PDF 91 KB)

Supplementary file7 (PDF 101 KB)

## Data Availability

The research material can be accessed by contacting the corresponding author.

## Footnotes

1 We used the same procedure to examine the functioning of alternative parent-report anxiety (≤ 24 items) and depression (≤ 9 items) subsets. The ROC curve analyses confirmed that the brief parent-report anxiety item set achieved an AUC of .84 which was not significantly different (*p* = .16) from the parent-report anxiety set that included the same items identified for adolescent-report of anxiety. The brief parent-report depression item set included the same five items as for adolescent-report of depression.

2 Sample sizes for adolescent boys and younger adolescents in the clinic-referred depression subsample were less than 30 (*n* = 17 and 21). The *post-hoc* power calculations determined that the results based on these sample sizes at the given AUC were still sufficiently powered.

## References

[CR1] Aebi M, Kuhn C, Banaschewski T, Grimmer Y, Poustka L, Steinhausen HC, Goodman R (2017). The contribution of parent and youth information to identify mental health disorders or problems in adolescents. Child and Adolescent Psychiatry and Mental Health.

[CR2] Albano AM, Silverman WK (1996). The Anxiety Disorders Interview Schedule for Children for DSM-IV: Clinician manual (Child and Parent Versions).

[CR3] American Psychiatric Association. (1994). *Diagnostic and Statistical Manual of Mental Disorders (4th ed.)*. Washington, DC: American Psychiatric Publishing.

[CR4] American Psychiatric Association (2000). Diagnostic and Statistical Manual for Mental Disorders-IV-TR.

[CR5] American Psychiatric Association. (2013). *Diagnostic and Statistical Manual of Mental Disorders (DSM-5)*. Washington, DC.: American Psychiatric Publishing.

[CR6] Angold A, Costello E (1987). Mood and Feelings Questionnaire (MFQ).

[CR7] Angold A, Costello EJ, Messer SC, Pickles A (1995). Development of a short questionnaire for use in epidemiological studies of depression in children and adolescents. International Journal of Methods in Psychiatric Research.

[CR8] Axelson DA, Birmaher B (2001). Relation between anxiety and depressive disorders in childhood and adolescence. Depression and Anxiety.

[CR9] Baptista MN, Borges L, Serpa ALDO (2017). Gender and Age-related Differences in Depressive Symptoms among Brazilian Children and Adolescents. Paidéia (Ribeirão Preto).

[CR10] Becker, A., Woerner, W., Hasselhorn, M., Banaschewski, T., & Rothenberger, A. (2004). Validation of the parent and teacher SDQ in a clinical sample. *European Child & Adolescent Psychiatry*, *13 Suppl 2*, II11–6. 10.1007/s00787-004-2003-5 10.1007/s00787-004-2003-5 15243781

[CR11] Chorpita BF, Moffitt CE, Gray J (2005). Psychometric properties of the Revised Child Anxiety and Depression Scale in a clinical sample. Behaviour Research and Therapy.

[CR12] Chorpita BF, Yim L, Moffitt C, Umemoto LA, Francis SE (2000). Assessment of symptoms of DSM-IV anxiety and depression in children: A revised child anxiety and depression scale. Behaviour Research and Therapy.

[CR13] Choudhury, M. S., Pimentel, S. S., & Kendall, P. C. (1998). Childhood Anxiety Disorders : Parent – Child ( Dis ) Agreement Using a Structured Interview for the DSM-IV, *42*(8), 957–964. 10.1097/01.CHI.0000046898.27264.A2 10.1097/01.CHI.0000046898.27264.A2 12874498

[CR14] Cummings CM, Caporino NE, Kendall PC (2014). Comorbidity of anxiety and depression in children and adolescents: 20 years after. Psychological bulletin.

[CR15] Deighton J, Croudace T, Fonagy P, Brown J, Patalay P, Wolpert M (2014). Measuring mental health and wellbeing outcomes for children and adolescents to inform practice and policy: a review of child self-report measures. Child and Adolescent Psychiatry and Mental Health.

[CR16] De Los Reyes A, Augenstein TM, Wang M, Thomas SA, Drabick DAG, Burgers DE, Rabinowitz J (2015). The validity of the multi-informant approach to assessing child and adolescent mental health. Psychological Bulletin.

[CR17] Department of Health, & Department of Education. (2017). *Transforming Children and Young People’s Mental Health Provision: a Green Paper*. https://assets.publishing.service.gov.uk/government/uploads/system/uploads/attachment_data/file/664855/Transforming_children_and_young_people_s_mental_health_provision.pdf

[CR18] Desousa DA, Salum GA, Isolan LR, Manfro GG (2013). Sensitivity and specificity of the Screen for Child Anxiety Related Emotional Disorders (SCARED): A community-based study. Child Psychiatry and Human Development.

[CR19] Donnelly A, Fitzgerald A, Shevlin M, Dooley B, Donnelly A, Fitzgerald A (2019). Investigating the psychometric properties of the revised child anxiety and depression scale ( RCADS ) in a non-clinical sample of Irish adolescents. Journal of Mental Health.

[CR20] Dowdy, E., Furlong, M., Eklund, K., Saeki, E., & Ritchey, K. (2010). Screening for Mental Health and Wellness: Current School-Based Practices and Emerging Possibilities. In B. Doll, W. Pfohl, & J. Yoon (Eds.), *Handbook of Youth Prevention Science* (p. 26). New York: Routledge. 10.4324/9780203866412

[CR21] Eaton DK, Lowry R, Brener ND, Grunbaum JA, Kann L (2004). Passive versus active parental permission in school-based survey research: Does the type of permission affect prevalence estimates of risk behaviors?. Evaluation Review.

[CR22] Ebesutani C, Bernstein A, Nakamura BJ, Chorpita BF, Weisz JR (2010). A psychometric analysis of the revised child anxiety and depression scale-parent version in a clinical sample. Journal of Abnormal Child Psychology.

[CR23] Ebesutani C, Korathu-Larson P, Nakamura BJ, Higa-McMillan C, Chorpita B (2017). The Revised Child Anxiety and Depression Scale 25–Parent Version: Scale Development and Validation in a School-Based and Clinical Sample. Assessment.

[CR24] Ebesutani C, Reise SP, Chorpita BF, Ale C, Regan J, Young J (2012). The Revised Child Anxiety and Depression Scale-Short Version: Scale reduction via exploratory bifactor modeling of the broad anxiety factor. Psychological Assessment.

[CR25] Essau C. A. (2003). Comorbidity of anxiety disorders in adolescents. *Depression and Anxiety*, *18*(1), 1–6. 10.1002/da.10107 10.1002/da.10107 12900947

[CR26] Essau CA (2008). Comorbidity of depressive disorders among adolescents in community and clinical settings. Psychiatry research.

[CR27] Evans R, Thirlwall K, Cooper P, Creswell C (2017). Using symptom and interference questionnaires to identify recovery among children with anxiety disorders. Psychological Assessment.

[CR28] Glover TA, Albers CA (2007). Considerations for evaluating universal screening assessments. Journal of School Psychology.

[CR29] Goodman, R. (1999). The extended version of the Strengths and Difficulties Questionnaire as a guide to child psychiatric caseness and consequent burden. *Journal of Child Psychology and Psychiatry, and Allied Disciplines*, *40*(5), 791–9. http://www.ncbi.nlm.nih.gov/pubmed/10433412 10433412

[CR30] Goodman R (2001). Psychometric properties of the strengths and difficulties questionnaire. Journal of the American Academy of Child and Adolescent Psychiatry.

[CR31] Goodman, R., Ford, T., Simmons, H., Gatward, R., & Meltzer, H. (2000). Using the Strengths and Difficulties Questionnaire (SDQ) to screen for child psychiatric disorders in a community sample. *The British Journal of Psychiatry : The Journal of Mental Science*, *177*, 534–539. 10.1192/bjp.177.6.534 10.1192/bjp.177.6.534 11102329

[CR32] Gyani A, Shafran R, Layard R, Clark DM (2013). Enhancing recovery rates: lessons from year one of IAPT. Behaviour research and therapy.

[CR33] Hammerton G, Zammit S, Potter R, Thapar A, Collishaw S (2014). Validation of a composite of suicide items from the Mood and Feelings Questionnaire (MFQ) in offspring of recurrently depressed parents. Psychiatry Research.

[CR34] Jiang S, Wang C, Weiss DJ (2016). Sample Size Requirements for Estimation of Item Parameters in the Multidimensional Graded Response Model. Frontiers in Psychology.

[CR35] Johnson SE, Lawrence D, Hafekost J, Saw S, Buckingham WJ, Sawyer M (2016). Service use by Australian children for emotional and behavioural problems: Findings from the second Australian Child and Adolescent Survey of Mental Health and Wellbeing. Australian & New Zealand Journal of Psychiatry.

[CR36] Jonge, T. De, Veenhoven, R., & Arends, L. (2014). Homogenizing Responses to Different Survey Questions on the Same Topic : Proposal of a Scale Homogenization Method Using a Reference Distribution, 275–300. 10.1007/s11205-013-0335-6 10.1007/s11205-013-0335-6 PMC3971463 24707072

[CR37] Kaufman J, Birmaher B, Axelson D, Perepletchikova F, Brent D, Ryan N (2016). K-SADS-PL.

[CR38] Kaufman J, Birmaher B, Brent D, Rao UMA, Flynn C, Moreci P (1997). Schedule for Affective Disorders and Schizophrenia for School-Age Children-Present and Lifetime Version (K-SADS-PL): Initial Reliability and Validity Data. Journal of the American Academy of Child and Adolescent Psychiatry.

[CR39] Kuo, E., Vander Stoep, A., McCauley, E., & Kernic, M. A. (2009). Cost-effectiveness of a school-based emotional health screening program. *The Journal of School Health, 79*(6), 277–285. PubMed. 10.1111/j.1746-1561.2009.00410.x 10.1111/j.1746-1561.2009.00410.x PMC2682225 19432868

[CR40] Kraemer HC (1992). Evaluating medical tests: Objective and Quantitative Guidelines.

[CR41] Kroenke K, Spitzer RL, Williams JBW (2001). The PHQ-9. Journal of general internal medicine.

[CR42] Kuhn C, Aebi M, Jakobsen H, Banaschewski T, Poustka L, Grimmer Y (2017). Effective Mental Health Screening in Adolescents: Should We Collect Data from Youth, Parents or Both?. Child psychiatry and human development.

[CR43] Lawrence, D., Johnson, S., Hafekost, J., Boterhoven de Haan, K., Sawyer, M., Ainley, J., & Zubrick, S. R. (2015). *The Mental Health of Children and Adolescents. Report on the Second Australian Child and Adolescent Survey of Mental Health and Wellbeing*. Canberra: Department of Health.

[CR44] LeDell, E., Petersen, M., & Van der Laan, M. (2014). cvAUC: Cross-Validated Area Under the ROC Curve Confidence Intervals. 10.1214/15-EJS1035 PMC4533123 26279737

[CR45] Lewis AJ, Bertino MD, Bailey CM, Skewes J, Lubman DI, Toumbourou JW (2014). Depression and suicidal behavior in adolescents : a multi-informant and multi-methods approach to diagnostic classification. Frontiers in Psychology.

[CR46] Liu C, Cox RB, Washburn IJ, Croff JM, Crethar HC (2017). The Effects of Requiring Parental Consent for Research on Adolescents’ Risk Behaviors: A Meta-analysis. Journal of Adolescent Health.

[CR47] Lyneham HJ, Abbott MJ, Rapee RM (2007). Interrater reliability of the anxiety disorders interview schedule for DSM-IV: Child and parent version. Journal of the American Academy of Child and Adolescent Psychiatry.

[CR48] Martel MM, Markon K, Smith GT (2017). Research Review: Multi-informant integration in child and adolescent psychopathology diagnosis. Journal of Child Psychology and Psychiatry, and Allied Disciplines.

[CR49] Merikangas KR, He JP, Brody D, Fisher PW, Bourdon K, Koretz DS (2010). Prevalence and Treatment of Mental Disorders Among US Children in the 2001–2004 NHANES. Pediatrics.

[CR50] Myers, K., & Winters, N. C. (2002). Ten-Year Review of Rating Scales. I: Overview of Scale Functioning, Psychometric Properties, and Selection. *Journal of the American Academy of Child and Adolescent Psychiatry*, *41*(2), 114–122. 10.1097/00004583-200202000-00004 10.1097/00004583-200202000-00004 11837400

[CR51] National Institute for Health and Care Excellence. (2019). Depression in children and y young oung people : identification and management, (June). www.nice.org.uk/guidance/ng134 31577402

[CR52] Orchard F, Pass L, Marshall T, Reynolds S (2017). Clinical characteristics of adolescents referred for treatment of depressive disorders. Child and Adolescent Mental Health.

[CR53] Peters, G. (2018). _userfriendlyscience: Quantitative analysis made accessible_. 10.17605/osf.io/txequ

[CR54] Piqueras, J. A., Martín-vivar, M., Sandin, B., San, C., & Pineda, D. (2017). Journal of A ff ective Disorders The Revised Child Anxiety and Depression Scale : A systematic review and reliability generalization meta-analysis. *Journal of Affective Disorders*, *218*(December 2016), 153–169. 10.1016/j.jad.2017.04.022 10.1016/j.jad.2017.04.022 28475961

[CR55] Polanczyk GV, Salum GA, Sugaya LS, Caye A, Rohde LA (2015). Annual research review: A meta-analysis of the worldwide prevalence of mental disorders in children and adolescents. Journal of Child Psychology and Psychiatry and Allied Disciplines.

[CR56] R Core Team. (2019). R: A language and environment for statistical computing. Vienna, Austria: R Foundation for Statistical Computing. https://www.r-project.org/

[CR57] Reardon, T., Spence, S. H., Hesse, J., Shakir, A., & Creswell, C. (2018). Identifying children with anxiety disorders using brief versions of the Spence Children's Anxiety Scale for children, parents, and teachers. *Psychological assessment, 30*(10), 1342–1355. 10.1037/pas0000570 10.1037/pas0000570 PMC6179143 29902050

[CR58] Revelle, W. (2018). psych: Procedures for Personality and Psychological Research. Evanston, Illinois, USA: Northwestern University. https://cran.r-project.org/package=psych

[CR59] Riegler A, Völkl-Kernstock S, Lesch O, Walter H, Skala K (2017). Attention deficit hyperactivity disorder and substance abuse: An investigation in young Austrian males. Journal of Affective Disorders.

[CR60] Robin X, Turck N, Hainard A, Tiberti N, Lisacek F, Sanchez J-C, Müller M (2011). pROC: an open-source package for R and S+ to analyze and compare ROC curves. BMC Bioinformatics.

[CR61] Roseman, M., Kloda, L. A., Saadat, N., Riehm, K. E., Ickowicz, A., Baltzer, F., Katz, L. Y., Patten, S. B., Rousseau, C., & Thombs, B. D. (2016). Accuracy of depression screening tools to detect major depression in children and adolescents: a systematic review. *Canadian Journal of Psychiatry. Revue Canadienne de Psychiatrie*, *61*(12), 746–757. PubMed. 10.1177/0706743716651833 10.1177/0706743716651833 PMC5564894 27310247

[CR62] Sadler K, Vizard Ti, Ford T, Goodman A, Goodman R, McManus S (2018). Mental Health of Children and Young People in England, 2017.

[CR63] Seligman LD, Ollendick TH (1998). Comorbidity of Anxiety and Depression in Children and Adolescents: An Integrative Review. Clinical Child and Family Psychology Review.

[CR64] Silverman WK, Saavedra LM, Pina AA (2001). Test-retest reliability of anxiety symptoms and diagnoses with the anxiety disorders interview schedule for DSM-IV: Child and parent versions. Journal of the American Academy of Child and Adolescent Psychiatry.

[CR65] Siu, A. L., & U S Preventive Services Task Force (2016). Screening for depression in adults: Us preventive services task force recommendation statement. JAMA.

[CR66] Spence SH (1997). Structure of anxiety symptoms among children: a confirmatory factor-analytic study. Journal of Abnormal Psychology.

[CR67] Spence SH (1998). A measure of anxiety symptoms among children. Behaviour research and therapy.

[CR68] Spence, S. H. (2018). Assessing anxiety disorders in children and adolescents, (3), 266–282. 10.1111/camh.12251 10.1111/camh.12251 32677290

[CR69] Spitzer RL, Kroenke K, Williams JBW, Lowe B (2006). A brief measure for assessing generalized anxiety disorder: the GAD-7. Archives of Internal Medicine.

[CR70] Stringaris A, Goodman R (2013). The value of measuring impact alongside symptoms in children and adolescents: a longitudinal assessment in a community sample. Journal of abnormal child psychology.

[CR71] Vander Stoep A, Mccauley E, Flynn C, Stone A (2009). Thoughts of Death and Suicide in Early Adolescence. Suicide and Lifethreatening Behavior.

[CR72] Villabø, M., Gere, M., Torgersen, S., March, J. S., & Kendall, P. C. (2012). Diagnostic efficiency of the child and parent versions of the Multidimensional Anxiety Scale for Children. *Journal of Clinical Child and Adolescent Psychology : The Official Journal for the Society of Clinical Child and Adolescent Psychology, American Psychological Association, Division 53*, *41*(1), 75–85. 10.1080/15374416.2012.632350 10.1080/15374416.2012.632350 22233247

[CR73] Whiteford HA, Degenhardt L, Rehm J, Baxter AJ, Ferrari AJ, Erskine HE (2013). Global burden of disease attributable to mental and substance use disorders: Findings from the Global Burden of Disease Study 2010. The Lancet.

[CR74] Wood, J. J., Piacentini, J. C., Bergman, R. L., McCracken, J., & Barrios, V. (2002). Concurrent validity of the anxiety disorders section of the Anxiety Disorders Interview Schedule for DSM-IV: child and parent versions. *Journal of Clinical Child and Adolescent Psychology: the Official Journal for the Society of Clinical Child and Adolescent Psychology, American Psychological Association, Division 53*, *31*(3), 335–342. 10.1207/S15374424JCCP3103_05 10.1207/S15374424JCCP3103_05 12149971

