# Supplementary material for: Using the 11-item Version of the RCADS to Identify Anxiety and Depressive Disorders in Adolescents
Source: Res Child Adolesc Psychopathol. 2021 Apr 1;49(9):1241–57. doi: 10.1007/s10802-021-00817-w (PMC8321965; doi:10.1007/s10802-021-00817-w)
Supplement: Supplementary file 1 — Supplementary file1 (PDF 86 KB) [file 10802_2021_817_MOESM1_ESM.pdf]

Using the 11-item Version of the RCADS to Identify Anxiety and Depressive Disorders in Adolescents

Journal of Abnormal Child Psychology

Electronic Supplementary Material 1: Rank-ordered item-total correlations and item-discrimination indices for the RCADS-P items

Item number	Item (RCADS Subscale)	Item-total	Item-anxiety/depression diagnosis correlation		
			Total	Controlled for Gender	Controlled for age
	Anxiety Items				
RCADS-P35	My child worries about what is going to happen (GAD)	.81*	.44*	.42*	.45*
RCADS-P1	My child worries about things (GAD)	.79*	.43*	.40*	.43*
RCADS-P22	My child worries that bad things will happen to him/her (GAD)	.76*	.43*	.41*	.45*
RCADS-P30	My child worries about making mistakes (SOC)	.76*	.43*	.40*	.42*
RCADS-P27	My child worries that something bad will happen to him/her (GAD)	.76*	.40*	.38*	.42*
RCADS-P45	My child worries when in bed at night (SEP)	.74*	.49*	.47*	.50*
RCADS-P32	My child worries what other people think of him/her (SOC)	.74*	.48*	.45*	.48*
RCADS-P43	My child feels afraid that he/she will make a fool of him/herself in front of people (SOC)	.74*	.49*	.46*	.48*
RCADS-P41	My child worries that he/she will suddenly get a scared feeling when there is nothing to be afraid of (PD)	.73*	.42*	.40*	.42*
RCADS-P20	My child worries about looking foolish (SOC)	.73*	.53*	.50*	.53*
RCADS-P34[a]	All of a sudden my child will feel really scared for no reason at all (PD)	.71*	.40*	.37*	.41*
RCADS-P4	My child worries when he/she thinks he/she has done poorly at something (SOC)	.70*	.35*	.32*	.34*
RCADS-P24	When my child has a problem, his/her heart beats really fast (PD)	.70*	.42*	.40*	.41*
RCADS-P7	My child feels scared when taking a test (SOC)	.70*	.36*	.33*	.36*
RCADS-P12	My child worries about doing badly at school work (SOC)	.69*	.41*	.38*	.40*
RCADS-P18	My child has trouble going to school in the mornings because of feeling nervous or afraid (SEP)	.69*	.45*	.44*	.47*
RCADS-P28	When my child has a problem, he/she feels shaky (PD)	.67*	.39*	.37*	.39*
RCADS-P8	My child worries when he/she thinks someone is angry with him/her (SOC)	.67*	.33*	.31*	.34*
RCADS-P33	My child is afraid of being in crowded places (like shopping centres, the cinema, buses, busy playgrounds) (SEP)	.66*	.41*	.39*	.42*
RCADS-P13	My child worries that something awful will happen to someone in the family (GAD)	.65*	.27*	.25*	.28*

RCADS-P3	When my child has a problem, he/she gets a funny feeling in his/her stomach (PD)	.63*	.41*	.38*	.41*
RCADS-P38	My child feels afraid if he/she has to talk in front of the class (SOC)	.63*	.38*	.36*	.38*
RCADS-P14[a]	My child suddenly feels as if he/she can't breathe when there is no reason for this (PD)	.61*	.31*	.28*	.30*
RCADS-P39[a]	My child's heart suddenly starts to beat too quickly for no reason (PD)	.61*	.38*	.35*	.37*
RCADS-P46	My child would feel scared if he/she had to stay away from home overnight (SEP)	.56*	.31*	.29*	.33*
RCADS-P37	My child thinks about death (GAD)	.55*	.28*	.26*	.29*
RCADS-P26[a]	My child suddenly start to tremble or shake when there is no reason for this (PD)	.54*	.29*	.27*	.28*
RCADS-P9	My child worries about being away from me (SEP)	.54*	.18*	.15*	.21*
RCADS-P5	My child feels afraid of being alone at home (SEP)	.51*	.23*	.20*	.24*
RCADS-P17[a]	My child feels scared to sleep on his/her own (SEP)	.47*	.22*	.20*	.26*
RCADS-P36[a]	My child suddenly become dizzy or faint when there is no reason for this (PD)	.43*	.22*	.19*	.21*
	Depression Items				
RCADS-P19	My child has no energy for things (MDD)	.83*	.42*	.41*	.39*
RCADS-P29	My child feels worthless (MDD)	.81*	.37*	.36*	.37*
RCADS-P21	My child is tired a lot (MDD)	.80*	.42*	.41*	.38*
RCADS-P2	My child feels sad or empty (MDD)	.78*	.40*	.39*	.39*
RCADS-P6	Nothing is much fun for my child anymore (MDD)	.76*	.39*	.38*	.37*
RCADS-P25	My child cannot think clearly (MDD)	.74*	.28*	.27*	.28*
RCADS-P11	My child has trouble sleeping (MDD)	.71*	.29*	.28*	.29*
RCADS-P40	My child feels like he/she doesn't want to move (MDD)	.69*	.30*	.28*	.28*
RCADS-P15	My child has problems with his/her appetite (MDD)	.68*	.29*	.27*	.27*
RCADS-P47	My child feel restless (MDD)	.67*	.25*	.24*	.23*

Note. GAD = generalised anxiety disorder, PD = panic disorder, SOC = social anxiety disorder, SEP = separation anxiety disorder, [a]Item > 80% of responses 'never' in a community sample, *p < .01
