# Supplementary material for: Using the 11-item Version of the RCADS to Identify Anxiety and Depressive Disorders in Adolescents
Source: Res Child Adolesc Psychopathol. 2021 Apr 1;49(9):1241–57. doi: 10.1007/s10802-021-00817-w (PMC8321965; doi:10.1007/s10802-021-00817-w)
Supplement: Supplementary file 2 — Supplementary file2 (PDF 99 KB) [file 10802_2021_817_MOESM2_ESM.pdf]

# Using the 11-item Version of the RCADS to Identify Anxiety and Depressive Disorders in Adolescents

*Journal of Abnormal Child Psychology*

Electronic Supplementary Material 2: ROC analyses for alternative combinations of adolescent-report anxiety items (≤12 items) and depression items (≤9 items) compared to original RCADS and RCADS-25.

| Scale | Number of items | Total | | | Boys | | Girls | | Older adolescents | | Younger adolescents | |
|---|---|---|---|---|---|---|---|---|---|---|---|---|
| | | AUC (total) | Cut-off | Sens/ Spec | Cut-off | Sens/ Spec | Cut-off | Sens/ Spec | Cut-off | Sens/ Spec | Cut-off | Sens/S pec |
| RCADS-47- Anxiety | 31 | .75 | 38.5 | .72/ .69 | 26.5 | .77/ .73 | 41.5 | .70/ .67 | 38.5 | .77/ .65 | 35.5 | .72/ .66 |
| RCADS-25 - Anxiety | 15 | .71 | 13.5 | .73/ .61 | 10.9 | .70/ .62 | 15.5 | .68/ .61 | 14.5 | .70/ .65 | 13.5 | .72/ .61 |
| Anx 1 | 12 | .79 | 15.5 | .73/ .72 | 8.5 | .82/ .72 | 17.5 | .72/ .68 | 15.5 | .79/ .71 | 14.5 | .73/ .68 |
| Anx 2 | 11 | .79 | 13.5 | .76/ .68 | 9.5 | .83/ .74 | 15.5 | .74/ .66 | 14.5 | .78/ .71 | 13.5 | .72/ .70 |
| Anx 3 | 10 | .80 | 12.5 | .75/ .72 | 7.5 | .81/ .72 | 13.5 | .74/ .67 | 13.5 | .77/ .75 | 11.5 | .76/ .68 |
| Anx 4 | 9 | .80 | 11.5 | .74/ .73 | 7.5 | .79/ .74 | 12.5 | .74/ .67 | 12.5 | .76/ .75 | 10.5 | .74/ .69 |
| Anx 5 | 8 | .80 | 10.5 | .77/ .72 | 7.5 | .79/ .77 | 11.5 | .75/ .66 | 10.5 | .83/ .70 | 9.5 | .79/ .69 |
| Anx 6 | 7 | .80 | 8.5 | .77/ .72 | 5.5 | .81/ .74 | 9.5 | .75/ .67 | 8.5 | .82/ .73 | 8.5 | .72/ .71 |
| **Anx 7** | **6** | **.81** | **7.5** | **.77/ .74** | **4.5** | **.83/ .75** | **8.5** | **.73/ .70** | **7.5** | **.81/ .78** | **7.5** | **.74/ .72** |
| Anx 8 | 5 | .81 | 5.5 | .76/ .73 | 3.5 | .86/ .75 | 6.5 | .76/ .70 | 5.5 | .79/ .75 | 5.5 | .72/ .71 |
| Anx 9 | 4 | .81 | 3.5 | .83/ .66 | 2.5 | .88/ .74 | 4.5 | .76/ .68 | 3.5 | .86/ .68 | 3.5 | .81/ .65 |
| Anx 10 | 3 | .81 | 2.5 | .88/ .71 | 2.5 | .85/ .78 | 3.5 | .77/ .69 | 3.5 | .77/ .74 | 2.5 | .87/ .62 |
| Anx 11 | 2 | .81 | 1.5 | .82/ .70 | 0.5 | .91/ .65 | 1.5 | .85/ .57 | 1.5 | .80/ .65 | 1.5 | .83/ .73 |

*Note.* Anx 1-11= alternative anxiety item sets, Sens/Spec = Sensitivity/Specificity.

| Scale | Number of items | Total | | | Boys | | Girls | | Older adolescents | | Younger adolescents | |
|---|---|---|---|---|---|---|---|---|---|---|---|---|
| | | AUC (total) | Cut-off | Sens/ Spec | Cut-off | Sens/ Spec | Cut-off | Sens/ Spec | Cut-off | Sens/ Spec | Cut-off | Sens/ Spec |
| RCADS-47 - Depression | 10 | .86 | 14.7 | .83/ .71 | 13.5 | .88/ .77 | 15.5 | .75/ .71 | 15.5 | .73/ .72 | 16.8 | .86/ .83 |
| RCADS-25 - Depression | 10 | .86 | 14.7 | .83/ .71 | 13.5 | .88/ .77 | 15.5 | .75/ .71 | 15.5 | .73/ .72 | 16.8 | .86/ .83 |
| Depr 1 | 9 | .86 | 13.5 | .79/ .74 | 12.5 | .80/ .80 | 13.5 | .79/ .68 | 13.5 | .77/ .68 | 14.5 | .80/ .79 |
| Depr 2 | 8 | .87 | 12.5 | .79/ .78 | 10.5 | 1/ .78 | 12.5 | .79/ .73 | 12.5 | .77/ .75 | 12.5 | .85/ .79 |
| Depr 3 | 7 | .87 | 10.5 | .81/ .74 | 9.5 | .94/ .80 | 10.5 | .81/ .69 | 10.5 | .79/ .69 | 10.5 | .85/ .77 |
| Depr 4 | 6 | .86 | 9.5 | .81/ .76 | 8.5 | .88/ .80 | 9.5 | .82/ .71 | 9.5 | .79/ .71 | 9.5 | .86/ .78 |
| **Depr 5** | **5** | **.87** | **8.5** | **.82/ .77** | **7.5** | **.88/ .83** | **8.5** | **.82/ .71** | **8.5** | **.79/ .70** | **8.5** | **.90/ .80** |
| Depr 6 | 4 | .86 | 5.5 | .89/ .70 | 5.5 | .88/ .82 | 5.5 | .89/ .63 | 5.5 | .86/ .64 | 6.5 | .90/ .82 |
| Depr 7 | 3 | .86 | 4.5 | .77/ .77 | 3.5 | .88/ .78 | 4.5 | .76/ .73 | 4.5 | .72/ .71 | 4.5 | .90/ .81 |
| Depr 8 | 2 | .84 | 2.5 | .83/ .70 | 2.5 | .88/ .77 | 2.5 | .80/ .66 | 2.5 | .80/ .62 | 2.5 | .90/ .74 |

*Note.* Depr 1- Depr 8 = Alternative depression item sets, Sens/Spec = Sensitivity/Specificity.
