# Supplementary material for: Using the 11-item Version of the RCADS to Identify Anxiety and Depressive Disorders in Adolescents
Source: Res Child Adolesc Psychopathol. 2021 Apr 1;49(9):1241–57. doi: 10.1007/s10802-021-00817-w (PMC8321965; doi:10.1007/s10802-021-00817-w)
Supplement: Supplementary file 3 — Supplementary file3 (PDF 162 KB) [file 10802_2021_817_MOESM3_ESM.pdf]

Using the 11-item Version of the RCADS to Identify Anxiety and Depressive Disorders in Adolescents

Journal of Abnormal Child Psychology

Electronic Supplementary Material 3: Examining the functioning of items assessing risk of suicide/self-harm and symptom impact and duration.

Adolescent-report

a) Rank-ordered item-discrimination indices for the suicidal ideation items

Item name	Item	Item-depression diagnosis correlation: Total	Controlled for Gender	Controlled for age
MFQ19_T	I thought about killing myself.	.39*	.39*	.40*
MFQ16_T	I thought that life wasn't worth living.	.36*	.35*	.35*
MFQ17_T	I thought about death or dying.	.32*	.32*	.33*
MFQ18_T	I thought my family would be better off without me.	.18*	.17*	.21*

Note. MFQ-P16-19_T = transformed MFQ items, *p < .01.

b) ROC curve analyses for 5 RCADS depression items with different combinations of suicidal ideation items

	Total			Boys		Girls		Older adolescents		Younger adolescents	
	AUC (total)	Cut-off	Sens/ Spec	Cut-off	Sens/ Spec	Cut-off	Sens/ Spec	Cut-off	Sens/ Spec	Cut-off	Sens/ Spec
5 Depression items	.87	8.5	.82/ .77	7.5	.88/ .83	8.5	.82/ .71	8.5	.79/ .70	8.5	.90/ .80
5 Depression items + MFQ 19_T, MFQ 16_T, MFQ 17_T	.88	11.25	.83/ .76	10.3	.92/ .82	11.25	.83/ .72	11.75	.80/ .78	11.25	.82/ .78
5 Depression items + MFQ 19_T, MFQ 16_T	.89	10.75	.80/ .79	10.25	.92/ .86	10.25	.83/ .73	10.75	.80/ .77	10.75	.82/ .81
**5 Depression items + MFQ 19_T**	**.89**	**9.25**	**.83/ .79**	**8.8**	**.92/ .85**	**9.25**	**.83/ .76**	**9.25**	**.80/ .78**	**9.25**	**.91/ .80**

Note. Sens/Spec = Sensitivity/Specificity. MFQ-P16-19_T = transformed MFQ-C items.

c) Summary of binary logistic regression for 5 RCADS depression items with and without suicidal ideation item

Model	*b (SE)*	Wald *z*-value	OR (95% CI)	McFadden's R^2	Model fit change (χ^2)
Model 1					
Constant	-5.73 (.58)	-9.83*		.34	143.81**
RCADS 5 depression items - A	.51 (.06)	8.55*	1.66		
Model 2					
Constant	-5.98 (.77)	-7.81*		.41	6.23 (p = .013)
RCADS 5 depression items - A	.45 (.08)	5.55*	1.56 [1.35-1.85]		
MFQ19_T	.45 (.18)	2.50 (p = .012)	1.56 [1.10 - 2.23]		

Note. MFQ-19_T = transformed MFQ-C item, A = adolescent-report, *p < .01.

d) Rank-ordered item-discrimination indices for the symptom impact and duration items

Item name	Item	Item-any anxiety/depressive disorder diagnosis correlation		
		Total	Controlled for Gender	Controlled for age
Impact - Family and Friends[a]	How much of an impact do these difficulties have on your family and friends?	.53*	.51*	.52*
Impact – Distress	How much do these difficulties upset or distress you?	.52*	.49*	.52*
Impact – School	How much do these difficulties get in the way of your everyday life in these areas (School)	.52*	.50*	.50*
Impact - Outside the school	How much do these difficulties get in the way of your everyday life in these areas (Outside the school)	.44*	.42*	.42*
Impact – Friends	How much do these difficulties get in the way of your everyday life in these areas (Friends)	.40*	.37*	.38*
Impact -Time	How long have you had these difficulties for?	.34*	.31*	.30*
Impact – Home	How much do these difficulties get in the way of your everyday life in these areas (Home)	.30*	.27*	.30*

Note. [a]Item with a high (>10%) proportion of missing responses, **p* < .01.

e) ROC curve analyses for 11 RCADS items with different combinations of symptom impact and duration items

	Total			Boys		Girls		Older adolescents		Younger adolescents	
	AUC (total)	Cut-off	Sens/ Spec	Cut-off	Sens/ Spec	Cut-off	Sens/ Spec	Cut-off	Sens/ Spec	Cut-off	Sens/ Spec
RCADS 11-item Total	.82	12.5	.80/ .71	8.5	.84/ .70	14.5	.72/ .68	14.5	.80/ .76	11.5	.78/ .66
RCADS 11-item Total + 5 Impact items	.84	22.5	.82/ .74	18.0	.88/ .78	24.5	.75/ .70	24.5	.84/ .78	21.5	.76/ .73
RCADS 11-item Total + 4 Impact items	.85	19.5	.83/ .73	16.5	.83/ .78	21.5	.77/ .71	22.5	.80/ .79	18.5	.76/ .70
RCADS 11-item Total + 3 Impact items	.84	18.5	.81/ .73	14.5	.88/ .80	20.5	.75/ .70	20.5	.84/ .79	17.5	.74/ .71
RCADS 11-item **Total + 2 Impact items**	**.84**	**16.5**	**.84/ .72**	**13.5**	**.88/ .78**	**18.5**	**.76/ .66**	**18.6**	**.86/ .74**	**16.5**	**.74/ .72**
RCADS 11-item Total + 1 Impact item	.83	14.5	.83/ .71	10.5	.89/ .71	17.5	.72/ .70	17.5	.79/ .79	14.5	.73/ .73

Note. Sens/Spec = Sensitivity/Specificity.

f) Summary of binary logistic regression for total scale with and without symptom impact items (adolescent-report)

Model		*b* (*SE*)	Wald *z*-value	OR (95% CI)	McFadden's R^2	Model fit change (χ^2)
Model 1						
	Constant	-2.44 (.28)	-8.81*		.28	142.47*
	RCADS 11 item Total - A	.19 (.02)	10.02*	1.21 [1.64, 1.25]		
Model 2						
	Constant	-3.97 (.44)	-9.00*		.41	26.59*

RCADS 11 item Total - A	.11 (.03)	4.37*	1.12 [1.07, 1.18]
Impact items - A	.62 (.13)	4.90*	1.85 [1.46, 2.40]

Note. A = adolescent-report, *$p < .01$.

Parent-report

a) Rank-ordered item-discrimination indices for the suicidal ideation items

		Item-depression diagnosis correlation		
Item name	Item	Total	Controlled for Gender	Controlled for age
MFQ-P16_T	My child thought that life wasn't worth living	.33*	.33*	.33*
MFQ-P17_T	My child thought about death or dying	.22*	.22*	.25*
MFQ-P19_T	My child thought about killing himself/herself	.21*	.21*	.22*
MFQ-P18_T	My child thought my family would be better off without him/her	0.05	0.04	0.08

Note. MFQ-P16-19_T = transformed MFQ-P item, **p* < .01.

b) ROC curve analyses for 5 RCADS items plus different combinations of suicidal ideation items

	Total			Boys		Girls		Older adolescents		Younger adolescents	
	AUC (total)	Cut-off	Sens/ Spec	Cut-off	Sens/ Spec	Cut-off	Sens/ Spec	Cut-off	Sens/ Spec	Cut-off	Sens/ Spec
5 Depression items	.86	6.5	.84/ .75	5.5	.93/ .75	6.5	.85/ .72	6.5	.82/ .74	6.5	.90/ .76
**5 Depression items + MFQ-P16_T**[1]	**.87**	**6.75**	**.90/ .77**	**6.5**	**.90/ .84**	**6.75**	**.90/ .74**	**6.5**	**.86/ .76**	**7.25**	**.90/ .84**
5 Depression items + MFQ-P19_T	.87	6.75	.92/ .76	6.5	.90/ .84	7.25	.79/ .78	6.5	.90/ .75	7.25	.90/ .82

Note. Sens/Spec = Sensitivity/Specificity, MFQ-P16-19_T = transformed MFQ-P item.

c) Summary of binary logistic regression for 5 RCADS depression items with and without suicidal ideation item

Model	*b (SE)*	Wald *z*-value (p-value)	OR (95% CI)	McFadden's R^2	Model fit change (χ^2)
Model 1					
Constant	-4.41 (.43)	-10.36*		.32	111.16*
RCADS 5 depression items - P	.43 (.05)	8.42*	1.66 [1.49, 1.89]		
Model 2					
Constant	-4.92 (.57)	-8.58*		.61	.451 (*p* = .502)
RCADS 5 depression items - P	.45 (.07)	6.43*	1.56 [1.38-1.81]		
MFQ-P19_T	.15 (.23)	.68 (*p* = .497)	1.17 [.73-1.81]		

Note. MFQ-P19_T = transformed MFQ-P item, P = parent-report, **p* < .01.

[1] The ROC curve analyses confirmed that the optimal parent-report depression scale with MFQ-16 risk item was not significantly different (*p* = .93) from the parent-report depression scale that included the same MFQ-19 risk item as adolescent-report depression scale.

d) Rank-ordered item-discrimination indices for the 11 RCADS items plus symptom impact and duration items

Item name	Item	Item- anxiety diagnosis correlation<br>Total	Controlled for Gender	Controlled for age
Impact-Distress	How much do these difficulties upset or distress your child?	.65*	.63*	.65*
Impact-Family	How much of an impact do these difficulties have on you or the family as a whole?	.60*	.58*	.61*
Impact-School	How much do these difficulties get in the way of your child's everyday life in these areas? (School)	.59*	.57*	.59*
Impact-Outside the school	How much do these difficulties get in the way of your child's everyday life in these areas? (Outside the school)	.58*	.56*	.58*
Impact-Home	How much do these difficulties get in the way of your child's everyday life in these areas? (Home)	.54*	.52*	.54*
Impact-Friends	How much do these difficulties get in the way of your child's everyday life in these areas? (Friends)	.49*	.46*	.49*
Impact-Time	How long has your child had these difficulties for?	.23*	.21*	.24*

Note. *$p < .01$.

e) ROC curve analyses for different combinations of symptom impact and duration items

		Total<br>Boys		Girls			Older adolescents			Younger adolescents	
	AUC (total)	Cut-off	Sens/ Spec	Cut-off	Sens/ Spec	Cut-off	Sens/ Spec	Cut-off	Sens/ Spec	Cut-off	Sens/ Spec
RCADS 11-itemTotal	.88	10.5	.80/ .79	7.5	.96/ .80	11.5	.78/ .75	9.5	.84/ .77	10.5	.80/ .77
RCADS 11-itemTotal – matched to adolescent-report	.89	14.5	.82/ .80	12.5	.86/ .84	14.5	.87/ .74	14.5	.84/ .83	14.5	.80/ .79
RCADS 11-itemTotal + 6 Impact items	.90	20.5	.84/ .79	17.5	.82/ .82	22.5	.83/ .78	20.5	.84/ .80	20.5	.85/ .79
RCADS 11-itemTotal + 5 Impact items	.90	19.5	.82/ .80	15.5	.89/ .84	21.5	.81/ .80	18.5	.87/ .80	19.6	.81/ .79
RCADS 11-itemTotal + 4 Impact items	.90	18.5	.81/ .81	14.5	.86/ .84	19.5	.83/ .77	17.5	.87/ .80	18.5	.80/ .80
RCADS 11-itemTotal + 3 Impact item	.90	16.5	.81/ .80	13.5	.86/ .84	17.5	.84/ .76	15.5	.86/ .80	16.5	.80/ .79
RCADS 11-itemTotal + 2 Impact item	.90	14.5	.82/ .80	11.5	.92/ .82	15.5	.81/ .76	13.5	.86/ .80	14.5	.82/ .79
**RCADS 11-itemTotal + 1 Impact item[2]**	**.89**	**12.5**	**.83/ .80**	**10.5**	**.84/ .84**	**13.5**	**.83/ .75**	**12.5**	**.84/ .83**	**12.5**	**.81/ .79**

Note. Sens/Spec = Sensitivity/Specificity.

[2] The ROC curve analyses confirmed that the optimal parent-report total scale with one symptom impact item was not significantly different ($p = .62$) from the parent-report total scale that included the same symptom impact items as adolescent-report total scale.

f) Summary of binary logistic regression for the 11 RCADS items with and without symptom impact items

Model	*b (SE)*	Wald *z*-value (*p*-value)	OR (95% CI)	McFadden's R^2	Model fit change (χ^2)
Model 1					
Constant	-2.74 (.28)	-9.94*	1.29 [1.24, 1.35]	.42	213.67*
RCADS 11 item Total - P	.26 (.02)	11.02*			
Model 2					
Constant	-3.79 (.40)	-9.42*		.50	17.06*
RCADS 11 item Total - P	.15 (.03)	4.52*	1.16 [1.09, 1.24]		
Impact items - P	.53 (.13)	4.02*	1.69 [1.31, 2.20]		

Note. P = parent-report, *$p < .01$.
