# Supplementary material for: Using the 11-item Version of the RCADS to Identify Anxiety and Depressive Disorders in Adolescents
Source: Res Child Adolesc Psychopathol. 2021 Apr 1;49(9):1241–57. doi: 10.1007/s10802-021-00817-w (PMC8321965; doi:10.1007/s10802-021-00817-w)
Supplement: Supplementary file 4 — Supplementary file4 (PDF 71 KB) [file 10802_2021_817_MOESM4_ESM.pdf]

Using the 11-item Version of the RCADS to Identify Anxiety and Depressive Disorders in Adolescents

Journal of Abnormal Child Psychology

Electronic Supplementary Material 4: McDonald's omega coefficients with 95% confidence intervals for 11 RCADS items/original RCADS and RCADS-25 anxiety/depression subscales and total scales – adolescent-report and parent-report.

	Community Sample	Clinic-referred sample		
		Total sample	Anxiety subsample	Depression subsample
RCADS 6 anxiety items - A	.89 [.87-.91]	.72 [.67-.78]	.73 [.67-.78]	.70 [.59-.80]
RCADS-Anxiety subscale - A	.97 [.96-.98]	.94 [.93-.95]	.94 [.93-.95]	.93 [.90-.95]
RCADS-25-Anxiety subscale - A	.92 [.90-.93]	.86 [.83-.88]	.85 [.82-.88]	.81 [.75-.88]
RCADS 5 depression items - A	.87 [.85-.90]	.88 [.85-.90]	.88 [.85-.90]	.72 [.62-.82]
RCADS-Depression subscale - A	.92 [.91-.94]	.90 [.99-.92]	.90 [.87-.92]	.82 [.76.-.88]
RCADS-25-Depression subscale - A	.92 [.91-.94]	.90 [.99-.92]	.90 [.87-.92]	.82 [.76.-.88]
RCADS 11 total items - A	.92 [.90-.93]	.83 [.80-.86]	.80 [.80-.87]	.74 [.65-.83]
RCADS-Total scale - A	.97 [.97-.98]	.95 [.94-.96]	.95 [.94-.96]	.92 [.89-.95]
RCADS-25-Total scale - A	.95 [.94-.96]	.89 [.87-.91]	.90 [.87-.92]	.84 [.78-.89]
RCADS 6 anxiety items - P	.90 [.88-.92]	.77 [.73-.82]	.77 [.72-.82]	.78 [.69-.86]
RCADS-Anxiety – subscale - P	.98 [.97-.98]	.94 [.92-.95]	.93 [.92-.95]	.93 [.90-.95]
RCADS-25-Anxiety subscale - P	.94 [.92-.95]	.85 [.81-.88]	.84 [.80-.87]	.82 [.75-.89]
RCADS 5 depression items - P	.90 [.88-.92]	.85 [.82-.88]	.85 [.82-.89]	.72 [.62-.82]
RCADS-Depression subscale - P	.94 [.93-.95]	.86 [.83-.89]	.86 [.83-.89]	.76 [.67-.84]
RCADS-25-Depression subscale - P	.94 [.93-.95]	.86 [.83-.89]	.86 [.83-.89]	.76 [.67-.84]
RCADS 11 total items - P	.94 [.92-.95]	.83 [.79-.86]	.84 [.80-.87]	.77 [.70-.85]
RCADS-Total scale- P	.97 [.96-.98]	.93 [.92-.94]	.93 [.92-.94]	.94 [.93-.95]
RCADS-25-Total scale - P	.97 [.96-.97]	.89 [.86-.91]	.89 [.87-.91]	.82 [.76-.89]

Note. A = adolescent-report, P = parent-report.
