# Supplementary material for: Using the 11-item Version of the RCADS to Identify Anxiety and Depressive Disorders in Adolescents
Source: Res Child Adolesc Psychopathol. 2021 Apr 1;49(9):1241–57. doi: 10.1007/s10802-021-00817-w (PMC8321965; doi:10.1007/s10802-021-00817-w)
Supplement: Supplementary file 5 — Supplementary file5 (PDF 79 KB) [file 10802_2021_817_MOESM5_ESM.pdf]

Using the 11-item Version of the RCADS to Identify Anxiety and Depressive Disorders in Adolescents

Journal of Abnormal Child Psychology

Electronic Supplementary Material 5: Convergent and divergent validity coefficients for RCADS 11 items/original RCADS-47/RCADS-25.

		MFQ-C/P - Clinic-referred Sample		
	MFQ-C/P Community Sample	Total sample	Anxiety subsample	Depression subsample
RCADS 6 anxiety items - A	.77*	.58*	.60*	.28 (*p* = .083)
RCADS-Anxiety subscale - A	.77*	.59*	.63*	.25 (*p* .111)
RCADS-25-Anxiety subscale - A	.76*	.55*	.59*	.20 (*p* = .189)
RCADS 5 depression items - A	.80*	.77*	.77*	.63*
RCADS-Depression subscale- A	.85*	.80*	.80*	.53*
RCADS-25-Depression subscale - A	.85*	.80*	.80*	.53*
RCADS 6 anxiety items - P	.76*	.50*	.53*	.44 (*p* = .011)
RCADS-Anxiety subscale - P	.77*	.49*	.52*	.50*
RCADS-25-Anxiety subscale - P	.76*	.48*	.50*	.46*
RCADS 5 depression items - P	.79*	.70*	.71*	.71*
RCADS-Depression subscale - P	.85*	.71*	.72*	.67*
RCADS-25-Depression subscale - P	.85*	.71*	.72*	.67*

Note. A = adolescent-report, P = parent-report, *p < .01.
