# Supplementary material for: Using the 11-item Version of the RCADS to Identify Anxiety and Depressive Disorders in Adolescents
Source: Res Child Adolesc Psychopathol. 2021 Apr 1;49(9):1241–57. doi: 10.1007/s10802-021-00817-w (PMC8321965; doi:10.1007/s10802-021-00817-w)
Supplement: Supplementary file 6 — Supplementary file6 (PDF 91 KB) [file 10802_2021_817_MOESM6_ESM.pdf]

Using the 11-item Version of the RCADS to Identify Anxiety and Depressive Disorders in Adolescents

Journal of Abnormal Child Psychology

Electronic Supplementary Material 6: ROC curve analyses for the 11-item RCADS-P (current study), the original RCADS-P, and the RCADS-25-P.

Number of items	RCADS: Anxiety			RCADS: Depression			RCADS: Total		
	6[a]	37[b]	15[c]	5[a]	10[b]	10[c]	11[a]	47[b]	25[c]
Total									
AUC	.86	.83	.82	.86	.84	.84	.88	.86	.86
Cut-off	5.5	34.4	10.4	6.5	11.625	11.625	10.5	41.4	19.3
Sensitivity/ Specificity	.80/.77	.73/.73	.74/.73	.84/.75	.81/.74	.81/.74	.80/.79	.78/.75	.77/.77
n (positive; negative)	197;227	210;227	211;225	76;364	77;373	77;373	206;214	223;214	232;214
Boys									
AUC	.90	.87	.84	.88	.85	.85	.91	.88	.88
Cut-off	4.5	27.5	7.8	5.5	9.5	9.5	7.5	34.7	12.50
Sensitivity/ Specificity	.84/.83	.81/.77	.76/.75	.93/.75	.86/.72	.86/.72	.96/.80	.82/.80	.92/.78
n (positive; negative)	44;93	48;93	46;93	14;129	14;130	14;130	45;91	50;91	50;91
Girls									
AUC	.83	.81	.8	.84	.84	.84	.86	.83	.84
Cut-off	6.5	37.5	11.9	6.5	12.25	12.25	11.5	45.5	21.5
Sensitivity/ Specificity	.75/.75	.75/.72	.74/.70	.85/.72	.79/.73	.79/.73	.78/.75	.76/.72	.75/.72
n (positive; negative)	153;134	162;134	165;132	62;235	63;243	63;243	179;117	173;123	182;123
Older adolescents									
AUC	.87	.84	.82	.84	.83	.83	.90	.86	.87
Cut-off	4.5	32.9	9.5	6.5	11.625	11.625	9.5	35.4	17.5
Sensitivity/ Specificity	.87/.71	.70/.70	.73/.70	.82/.74	.80/.73	.80/.73	.84/.77	.87/.71	.83/.73
n (positive; negative)	98;77	105;77	106;76	56;129	56;131	56;131	109;66	116;66	120;66
Younger adolescents									
AUC	.85	.84	.83	.89	.88	.88	.88	.86	.87
Cut-off	5.5	35.5	10.9	6.5	12.25	12.25	10.5	39.5	19.4
Sensitivity/ Specificity	.82/.77	.76/.76	.77/.74	.90/.76	.81/.76	.81/.76	.80/.77	.82/.73	.78/.78
n (positive; negative)	99;150	105;150	105;149	20;235	21;242	21;242	97;148	107;148	112;148

Note. [a]11-item RCADS-P, [b]RCADS-P, [c]RCADS-25-P.
