# Supplementary material for: Using the 11-item Version of the RCADS to Identify Anxiety and Depressive Disorders in Adolescents
Source: Res Child Adolesc Psychopathol. 2021 Apr 1;49(9):1241–57. doi: 10.1007/s10802-021-00817-w (PMC8321965; doi:10.1007/s10802-021-00817-w)
Supplement: Supplementary file 7 — Supplementary file7 (PDF 101 KB) [file 10802_2021_817_MOESM7_ESM.pdf]

Using the 11-item Version of the RCADS to Identify Anxiety and Depressive Disorders in Adolescents

Journal of Abnormal Child Psychology

Electronic Supplementary Material 7: Anxiety and depression screening items with instructions

Brief 11-item version of the RCADS for Adolescents
(adolescent-report)

Please put a circle around the word that shows how often each of these things happens to you. There are no right or wrong answers.

1. I have trouble going to school in the mornings because I feel nervous or afraid	Never	Sometimes	Often	Always
2. I have no energy for things	Never	Sometimes	Often	Always
3. I worry when I go to bed at night	Never	Sometimes	Often	Always
4. I worry about what is going to happen	Never	Sometimes	Often	Always
5. Nothing is much fun anymore	Never	Sometimes	Often	Always
6. All of a sudden I feel really scared for no reason at all	Never	Sometimes	Often	Always
7. I feel worthless	Never	Sometimes	Often	Always
8. I feel sad or empty	Never	Sometimes	Often	Always
9. When I have a problem, my heart beats really fast	Never	Sometimes	Often	Always
10. I am tired a lot	Never	Sometimes	Often	Always
11. I worry I might look foolish	Never	Sometimes	Often	Always

Impact supplement (optional):

Now please answer the following questions about those things that you said happen to you ‘sometimes’ ‘often’ or ‘always’.

How much do these difficulties upset or distress you?	Not at all	Only a little	Quite a lot	A great deal
How much do these difficulties get in the way of your everyday life in school?	Not at all	Only a little	Quite a lot	A great deal

Scoring:

Never/not at all = 0<br>Sometimes/only a little = 1<br>Often/quite a lot = 2<br>Always/a great deal = 3

Anxiety scale (summarise items 1, 3, 4, 6, 9, 11): ____

Depression scale (summarise items 2, 5, 7, 8, 10): ____

Total scale (summarise all items): ____

Total scale + impact (total scale + impact items): ____

Interpretation/cut-off scores:

		Boys	Girls
Anxiety Scale	Symptoms	≥ 5	≥ 9
Depression Scale	Symptoms	≥ 8	≥ 9
Total Scale	Symptoms	≥ 9	≥ 14
	Symptoms + Impact	≥ 14	≥ 18

Brief 11-item version of the RCADS for Adolescents
(parent-report)

Please put a circle around the word that shows how often each of these things happens for your child.

1. My child has trouble going to school in the mornings because of feeling nervous or afraid	Never	Sometimes	Often	Always
2. My child has no energy for things	Never	Sometimes	Often	Always
3. My child worries when in bed at night	Never	Sometimes	Often	Always
4. My child worries about what is going to happen	Never	Sometimes	Often	Always
5. Nothing is much fun for my child anymore	Never	Sometimes	Often	Always
6. All of a sudden my child will feel really scared for no reason at all	Never	Sometimes	Often	Always
7. My child feels worthless	Never	Sometimes	Often	Always
8. My child feels sad or empty	Never	Sometimes	Often	Always
9. When my child has a problem, his/her heart beats really fast	Never	Sometimes	Often	Always
10. My child is tired a lot	Never	Sometimes	Often	Always
11. My child worries about looking foolish	Never	Sometimes	Often	Always

Impact supplement (optional):

Now please answer the following questions about those things that you said happen to your child ‘sometimes’ ‘often’ or ‘always’.

How much do these difficulties upset or distress your child?	Not at all	Only a little	Quite a lot	A great deal
How much do these difficulties get in the way of your child’s everyday life in school?	Not at all	Only a little	Quite a lot	A great deal

Scoring:

Never/not at all = 0<br>Sometimes/only a little = 1<br>Often/quite a lot = 2<br>Always/a great deal = 3

Anxiety scale (summarise items 1, 3, 4, 6, 9, 11): ____

Depression scale (summarise items 2, 5, 7, 8, 10): ____

Total scale (summarise all items): ____

Total scale + impact (total scale + impact items): ____

Interpretation/cut-off scores:

		Boys	Girls
Anxiety Scale	Symptoms	≥ 5	≥ 7
Depression Scale	Symptoms	≥ 6	≥ 7
Total Scale	Symptoms	≥ 8	≥ 11
	Symptoms + Impact	≥ 13	≥ 15
